# Characterising paediatric mortality during and after acute illness in Sub-Saharan Africa and South Asia: a secondary analysis of the CHAIN cohort using a machine learning approach

**DOI:** 10.1016/j.eclinm.2023.101838

**Published:** 2023-02-06

**Authors:** The Childhood Acute Illness and Nutrition (CHAIN) Network, Abdoulaye Hama Diallo, Abu Sadat Mohammad Sayeem Bin Shahid, Ali Fazal Khan, Ali Faisal Saleem, Benson O. Singa, Blaise Siezanga Gnoumou, Caroline Tigoi, Catherine Achieng Otieno, Celine Bourdon, Chris Odhiambo Oduol, Christina L. Lancioni, Christine Manyasi, Christine J. McGrath, Christopher Maronga, Christopher Lwanga, Daniella Brals, Dilruba Ahmed, Dinesh Mondal, Donna M. Denno, Dorothy I. Mangale, Emmanuel Chimezi, Emmie Mbale, Ezekiel Mupere, Gazi Md Salauddin Mamun, Issaka Ouedraogo, George Githinji, James A. Berkley, Jenala Njirammadzi, John Mukisa, Johnstone Thitiri, Jonas Haggstrom, Joseph D. Carreon, Judd L. Walson, Julie Jemutai, Kirkby D. Tickell, Lubaba Shahrin, MacPherson Mallewa, Md. Iqbal Hossain, Mohammod Jobayer Chisti, Molly Timbwa, Moses Mburu, Moses M. Ngari, Narshion Ngao, Peace Aber, Philliness Prisca Harawa, Priya Sukhtankar, Robert H.J. Bandsma, Roseline Maimouna Bamouni, Sassy Molyneux, Sergey Feldman, Shalton Mwaringa, Shamsun Nahar Shaima, Syed Asad Ali, Syeda Momena Afsana, Syera Banu, Tahmeed Ahmed, Wieger P. Voskuijl, Zaubina Kazi

**Affiliations:** The CHAIN Network, KWTRP, 197 Lenana Place, Off Lenana Road, 43640 – 00100, Nairobi, Kenya

**Keywords:** Paediatric mortality, Wasting, Malnutrition, Post-discharge mortality, Explainable machine learning

## Abstract

**Background:**

A better understanding of which children are likely to die during acute illness will help clinicians and policy makers target resources at the most vulnerable children. We used machine learning to characterise mortality in the 30-days following admission and the 180-days after discharge from nine hospitals in low and middle-income countries (LMIC).

**Methods:**

A cohort of 3101 children aged 2–24 months were recruited at admission to hospital for any acute illness in Bangladesh (Dhaka and Matlab Hospitals), Pakistan (Civil Hospital Karachi), Kenya (Kilifi, Mbagathi, and Migori Hospitals), Uganda (Mulago Hospital), Malawi (Queen Elizabeth Central Hospital), and Burkina Faso (Banfora Hospital) from November 2016 to January 2019. To record mortality, children were observed during their hospitalisation and for 180 days post-discharge. Extreme gradient boosted models of death within 30 days of admission and mortality in the 180 days following discharge were built. Clusters of mortality sharing similar characteristics were identified from the models using Shapley additive values with spectral clustering.

**Findings:**

Anthropometric and laboratory parameters were the most influential predictors of both 30-day and post-discharge mortality. No WHO/IMCI syndromes were among the 25 most influential mortality predictors of mortality. For 30-day mortality, two lower-risk clusters (N = 1915, 61%) included children with higher-than-average anthropometry (1% died, 95% CI: 0–2), and children without signs of severe illness (3% died, 95% CI: 2–4%). The two highest risk 30-day mortality clusters (N = 118, 4%) were characterised by high urea and creatinine (70% died, 95% CI: 62–82%); and nutritional oedema with low platelets and reduced consciousness (97% died, 95% CI: 92–100%). For post-discharge mortality risk, two low-risk clusters (N = 1753, 61%) were defined by higher-than-average anthropometry (0% died, 95% CI: 0–1%), and gastroenteritis with lower-than-average anthropometry and without major laboratory abnormalities (0% died, 95% CI: 0–1%). Two highest risk post-discharge clusters (N = 267, 9%) included children leaving against medical advice (30% died, 95% CI: 25–37%), and severely-low anthropometry with signs of illness at discharge (46% died, 95% CI: 34–62%).

**Interpretation:**

WHO clinical syndromes are not sufficient at predicting risk. Integrating basic laboratory features such as urea, creatinine, red blood cell, lymphocyte and platelet counts into guidelines may strengthen efforts to identify high-risk children during paediatric hospitalisations.

**Funding:**

10.13039/100000865Bill & Melinda Gates FoundationOPP1131320.

**Research in context**

**Evidence before this study**

Despite implementation of WHO guidelines, recently published systematic reviews have shown that some groups of children in sub-Saharan Africa and Asia experience inpatient mortality rates of 15–25%, with similar subgroups of high-risk children suffering up to 20% mortality in the six months following hospital discharge. To understand the factors that identify these high-risk groups across different settings and disease-syndromes, we searched PubMed on February 2nd 2022 using the terms (“paediatric hospitalisation” or “paediatric post-discharge”) and “mortality” for articles published in English, French or Spanish, assessing the causes and predictors of acute and post-discharge paediatric mortality during acute illnesses in sub-Saharan Africa and Asia. Fifty of 137 studies identified contained relevant data, but no studies explored risk factor across multiple settings and different disease-syndromes categories. Additional, very few studies employed machine learning techniques, which can reveal previously unseen or underappreciated patterns of mortality.

**Added value of this study**

We used machine learning to identify clusters of mortality and found the most influential predictors of mortality in the 30-days following admission to hospital, and the 180 days after discharge, were anthropometric measurements and routine haematological and biochemical tests. The three highest risk clusters derived from the 30-day model were defined by nutritional oedema without signs of sepsis or severe illness, high serum urea and creatinine, and nutritional oedema with signs of sepsis or severe illness. Among children who were discharged, the highest risk clusters were defined by longer than average length of stay, leaving against medical advice, and severely poor anthropometric status at discharge. No WHO/IMCI clinical syndromes were among the 25 most influential predictors of mortality in either model.

**Implications of all the available evidence**

Most children admitted to hospital in this study survived when managed with current guidelines. However, subgroups of children with severe wasting, nutritional oedema, signs of sepsis, or evidence of renal insufficiency experienced extremely high rates of mortality. Developing novel interventions to treat these groups is a potentially important alternative to continued investment in incremental changes to the current WHO syndromic management approach. Consistent access to biochemical and haematological testing may strengthen efforts to identify high-risk children during paediatric hospitalisations.

## Introduction

Despite reductions in under-five mortality, the vast majority of child deaths still occur in low-and middle-income countries (LMIC).^1^ Children in these settings have a high risk of death during inpatient admission and for six to twelve months following discharge.^2^^,^^3^ The Childhood Acute Illness and Nutrition (CHAIN) Cohort is a study of children admitted to urban and rural hospitals in sub-Saharan Africa and south Asia.^4^^,^^5^ The cohort's primary analysis used survival analysis and structural equation modelling to estimate causal pathways to death based on the UNICEF framework for child mortality.^5^ That analysis demonstrated that the proportion of deaths in the six months following hospitalisation approximated inpatient deaths, that anthropometry captures a broad range of pathways to mortality, and that caregiver employment and mental health were directly associated with post-discharge mortality.

Rigorous causal modelling is an ideal approach to test investigator driven hypotheses but it may overlook important risk phenotypes not previously identified in the literature or the model's conceptual framework. Machine learning (ML) minimises the number of analytic decisions based on expert opinion or previous literature, but has traditionally focused on predicting outcomes rather than characterizing risks.^6^ However, model explanation tools have made it possible to form clusters of patients who the model predicts to be at similar risk of an outcome due to shared underlying characteristics. These explainable machine learning approach have been used to identify novel risk phenotypes in adult intensive care units, chronic obstructive pulmonary disorders, severe influenza admissions, and breast cancer,^7^, ^8^, ^9^, ^10^ but there are few examples of these methods being applied to paediatric care in LMICs.^11^, ^12^, ^13^ We aimed to describe phenotypes associated with mortality and survival based on the clinical, anthropometric, laboratory, and sociodemographic features using ML techniques within the CHAIN cohort.

## Methods

### Study design and participants

A description of the CHAIN Cohort has been published previously.^4^ We recruited children aged 2–23 months at admission to hospitals in Dhaka and Matlab (Bangladesh); Karachi (Pakistan); Kilifi, Nairobi and Migori (Kenya); Kampala (Uganda); Blantyre (Malawi); and Banfora (Burkina Faso) from November 2016 to January 2019. Children with traumatic injuries or conditions requiring surgery in the next six months were excluded. Each site oversampled children at high risk of mortality by stratifying enrolment by mid-upper arm circumference (MUAC) in the following ratio: two children with severe wasting or nutritional oedema (MUAC <11.5 cm if ≥6-months of age, MUAC <11.0 cm if <6-months of age, or bilateral pitting oedema), two with moderate wasting (MUAC <12.5 cm but ≥11.5 cm if ≥6-months of age, MUAC <12.0 cm but ≥11.0 cm if <6-months of age), and one with no wasting per week. Detailed clinical, anthropometric and sociodemographic information was collected at admission and discharge. Bedside malaria testing (CARESTART or SD Bioline Ag Pf-Pan) and HIV-1/2 testing (Alere 2 Determine or Uni-gold) were performed at admission. Complete blood counts and biochemical analysis were performed on admission and discharge samples by local clinical laboratories. Fieldworkers also visited participants’ home to collect global positioning system coordinates which were merged with geospatial datasets to calculate distance to the nearest health facility, and the admitting facility, and the population density in the square kilometre surrounding the household.^14^ Outcomes, including mortality, were ascertained from enrolment to 180 days after discharge from the index hospitalisation. Ethical approval for this study was obtained from the University of Oxford and all relevant site specific committees.^4^^,^^5^ Informed consent was given by the caregivers of all participants, and this manuscript was written in compliance with the STROBE and TRIPOD reporting guidelines.

### Statistical analysis

Data from all children enrolled in the CHAIN cohort were included in this analysis. We analysed mortality during two time periods, reflecting the primary epidemiological analysis of CHAIN.^5^ The first analysis assessed mortality in the 30-days following admission to hospital, the second evaluated mortality in the six-months following index hospital discharge.

Potential predictors of 30-day mortality included 207 admission variables (7 demographic, 184 clinical, 16 laboratory; Appendix 1). Clinical variables included a World Health Organisation/Integrated Management of Childhood Illness (WHO/IMCI) syndromic diagnoses for diarrhea, malaria, and pneumonia. Z-scores for weight-for-age (WAZ), and height-for-age (HAZ), and weight-for-height (WHZ) were calculated using the WHO AnthroPlus package. Many social variables were collected from caregivers after the child was medically stable, typically 48-h after admission. Consequently, social variables were not included in the 30-day model as they were missing in early deaths and missing data is used as a potential predictor by extreme gradient boosted models (XGBoost). The post-discharge model included 556 admission and discharge variables (11 demographic, 320 clinical, 39 laboratory and 186 socioeconomic, Appendix 1).

**Fig. 1:**
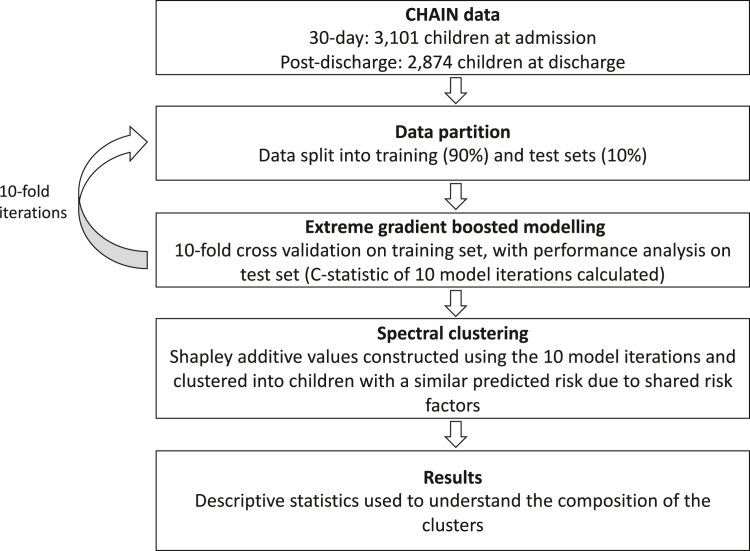
**Modelling pipeline applied to data from the Childhood Acute Illness & Nutrition (CHAIN) Cohort.** AUC—area under the curve.

Data for both analyses were randomly split into a training (90%) and test sets (10%). XGBoost Cox proportional hazards models were fit in the training sets with ten-fold cross-validation (Fig. 1).^15^^,^^16^ This model allows for participant censoring while also leveraging XGBoost's highly flexible ensemble approach.^7^ To account for the stratified recruitment, inverse-probability weights for selection were included (Appendix 2). Model performance was measured on the test sets using the C-statistic which approximates the area under the curve (AUC). This process was repeated ten times, resulting in ten models and ten test set performances. The ensemble of these ten XGBoost models are further referred to as the final XGBoost model.

To interpret the XGBoost model, and expose the contribution of each variable to each child's predicted risk, we calculated SHapley Additive exPlanations (SHAP) values.^17^ The 25 variables that provided the most information to the model were graphed. Variables outside the top 25 were found to make inconsequential contributions to the model. To understand if a simpler model would have similar predictive performance, we repeated the XGBoost estimation of the C-statistic using a decreasing number of predictors, beginning with the most influential 50 predictors, and iteratively removing the least informative remaining variable, re-estimating the C-statistic, and repeating this process until only one variable remained.

Using the SHAP values derived from the final XGBoost model, we clustered children at a similar predicted mortality risk due to shared underlying risk factors. Spectral clustering is superior to other clustering methods across varied scenarios,^18^ but the number of clusters to be identified by the algorithm is specified by the user. Preliminary data analyses suggested that six clusters contained discrete groups of very low and very high-risk children. Sensitivity analyses in which the final XGBoost models remained constant, but the spectral clustering algorithm generated four, five, six, seven and eight clusters were conducted.

The number of children, the Kaplan Meier estimated cumulative mortality, and median time-to-death for each cluster was estimated during the observed time period. Finally, the distributions of the most influential predictors of mortality across these clusters were described using Cohen's D values. Cohen's D compares the difference in mean values of variable *x* in Cluster_i_ to the mean values of variable *x* in all other clusters, over the standard deviation of variable *x* in the whole sample. These results are the mean difference between Cluster_i_ and all other Clusters expressed as standard deviations (SD). Six common clinical variables were added to these influential predictors to aid interpretation of the clusters (HIV status, malaria rapid test result, presence of oedema, consciousness level, caregiver reported diarrhoea, diagnosis of sepsis (per clinician), left hospital against medical advice).

The XGBoost model, SHAP values, and spectral clustering analyses were conducted in Python (v3.6.10), descriptive statistics were computed in R (v3.6.2, R Foundation for Statistical Computing). Additional rationale for the above analytic choices are available in Appendix 1.

### Role of the funding source

The funder of the study had no role in study design, data collection, data analysis, data interpretation, or writing of the report. Four authors (KDT, NN, SF, GG) had access to the data and were responsible for the decision to submit for publication.

## Results

The CHAIN cohort included 3101 children. The median age was 10.8 months (inter-quartile range [IQR]: 6.8, 15.7). Characteristics at admission to hospital are provided in Table 1, and discharge characteristics are in Appendix 3, Table S1. There were 350 deaths (11%) and 116 children (3.7%) were lost to follow-up.

**Table 1:** Patient characteristics at admission.

	Strata			Total
	NW (N = 1120)	MW (N = 763)	SWK (N = 1218)	N = 3101
**Demographics**				
Age — months median (IQR)	11.3 (7.2–16.2)	10.6 (7.0–14.8)	10.4 (6.4–15.8)	10.8 (6.8–15.7)
Sex (female) — no. (%)	432 (39)	329 (43)	583 (48)	1344 (43)
Clinical presentation at admission				
SIRS — no. (%)	395 (35)	284 (37)	386 (32)	1065 (34)
Severe Pneumonia — no. (%)	286 (26)	179 (23)	202 (17)	667 (22)
Neurological (AVPU > A) — no. (%)	46 (4.1)	47 (6.2)	53 (4.4)	146 (4.7)
Diarrhea — no. (%)	525 (47)	484 (63)	712 (58)	1721 (56)
Malaria (RDT positive) — no. (%)	202 (18)	106 (14)	132 (11)	440 (14)
Anemia — no. (%)^a^				
None	243 (22)	131 (17)	216 (18)	590 (19)
Mild	279 (25)	164 (21)	241 (20)	684 (22)
Moderate/severe	598 (53)	468 (61)	761 (62)	1827 (59)
Blood glucose — no. (%)^b^				
Normal blood glucose	1041 (93)	715 (94)	1101 (90)	2857 (92)
Abnormal blood glucose	79 (7.1)	48 (6.3)	117 (9.6)	244 (7.9)
Anthropometry at admission				
Weight-for-length z score — mean (sd)^c^	−0.6 (1.2)	−2.4 (1.0)	−3.1 (1.6)	−2.0 (1.7)
Weight-for-age z score — mean (sd)^c^	−1.1 (1.1)	−2.7 (0.9)	−4.0 (1.4)	−2.6 (1.7)
Length-for-age z score — mean (sd)	−1.1 (1.3)	−1.9 (1.3)	−3.1 (1.7)	−2.1 (1.7)
Small birth size — no. (%)^d^	132 (12)	132 (17)	245 (20)	509 (16)
HIV — no. (%)				
Negative	1010 (90)	693 (91)	1034 (85)	2737 (88)
Untested/refused	39 (3.5)	14 (1.8)	23 (1.9)	76 (2.5)
HIV infected	15 (1.3)	17 (2.2)	74 (6.1)	106 (3.4)
HIV exposed	56 (5.0)	39 (5.1)	87 (7.1)	182 (5.9)
Chronic conditions — no. (%)^e^	59 (5.3)	51 (6.7)	93 (7.6)	203 (6.6)
Prior hospitalization — no. (%)				
No prior admission	868 (78)	580 (76)	860 (71)	2308 (74)
<1 week	50 (4.5)	52 (6.8)	73 (6.0)	175 (5.6)
1 weeks to 1 month ago	56 (5.0)	42 (5.5)	109 (9.0)	207 (6.7)
>1month ago	146 (13)	89 (12)	176 (14)	411 (13)
**Caregiver characteristics**				
Biological mother as primary caregiver — no. (%)	1091 (97)	731 (96)	1134 (93)	2956 (95)
Mother sick — no. (%)	152 (14)	138 (18)	158 (13)	448 (14)
Caregiver education — no. (%)				
None	236 (21)	220 (29)	345 (28)	801 (26)
Primary	486 (43)	305 (40)	533 (44)	1324 (43)
Secondary/Tertiary	398 (35)	238 (31)	340 (28)	976 (31)
Maternal mental health (PHQ9) score — no. (%)				
None to Mild	970 (87)	614 (80)	933 (77)	2517 (81)
Moderate to severe	150 (13)	149 (20)	285 (23)	584 (19)
Mother working — no. (%)				
Self-employed	245 (22)	133 (17)	258 (21)	636 (21)
Employed	100 (8.9)	80 (10)	113 (9.3)	293 (9.5)
No reported income	775 (69)	550 (72)	847 (70)	2172 (70)
**Household-level exposures**				
Population density (/1000 square KM) median (IQR)	4.4 (0.4–13.7)	4.0 (0.7–19.1)	5.1 (0.4–18.8)	4.6 (0.5–16.4)
Assets index — no. (%)				
Quintile 1 (Least assets)	256 (23)	169 (22)	208 (17)	633 (20)
Quintile 2	221 (20)	156 (20)	252 (21)	629 (20)
Quintile 3	213 (19)	153 (20)	268 (22)	634 (21)
Quintile 4	215 (19)	141 (18)	240 (20)	596 (19)
Quintile 5 (Most assets)	215 (19)	144 (19)	250 (21)	609 (20)
Household food insecurity — no. (%)				
Low	713 (64)	473 (62)	659 (54)	1845 (60)
Medium	302 (27)	186 (24)	333 (27)	821 (26)
High	105 (9.4)	104 (13)	226 (18)	435 (14)
Improved toilet — no. (%)	835 (75)	606 (79)	900 (74)	2341 (75)
Improved water source — no. (%)	946 (84)	632 (83)	986 (81)	2564 (83)
Distance to the nearest health facility (km) median (IQR)	1.3 (0.5–3.1)	1.2 (0.5–3.7)	1.3 (0.5–2.8)	1.3 (0.5–3.1)
Means of travel to hospital — no. (%)				
Bus/ambulance/car/train	505 (45)	315 (41)	606 (50)	1426 (46)
Walking/Motorcycle/Tukutuku/rickshaw	615 (55)	448 (59)	612 (50)	1675 (54)
Travel cost to study hospital — no. (%)				
<1 US dollar	463 (41)	269 (35)	439 (36)	1171 (38)
1 to 5 US dollars	573 (51)	402 (53)	669 (55)	1644 (53)
≥5 US dollars	84 (7.5)	92 (12)	110 (9.0)	286 (9.2)
Travel time to study hospital — no. (%)				
<1 h	526 (47)	316 (41)	447 (37)	1289 (42)
1–2 h	414 (37)	272 (36)	457 (38)	1143 (37)
≥2 h	180 (16)	175 (23)	314 (26)	669 (21)

IQR; Interquartile range, SIRS; Systemic inflammatory response syndrome, AVPU; Alert, Voice, pain and Unresponsive, RDT; rapid diagnostic test, MUAC; mid-upper arm circumference.

a None: >11, Mild:10 to11, Moderate: 7 to 10 and Severe: <7.0 g/L.

b Abnormal blood glucose defined as blood glucose <3 or 10 mmol/dl.

c Children with oedema excluded from these values.

d Small birth size was defined as reported low birth weight (<2.5 kg) or born premature.

e Chronic conditions includes thalassemia, cerebral palsy, sickle cell disease, congenital cardiac disease and know TB (on treatment).

### 30-day mortality

**Fig. 2:**
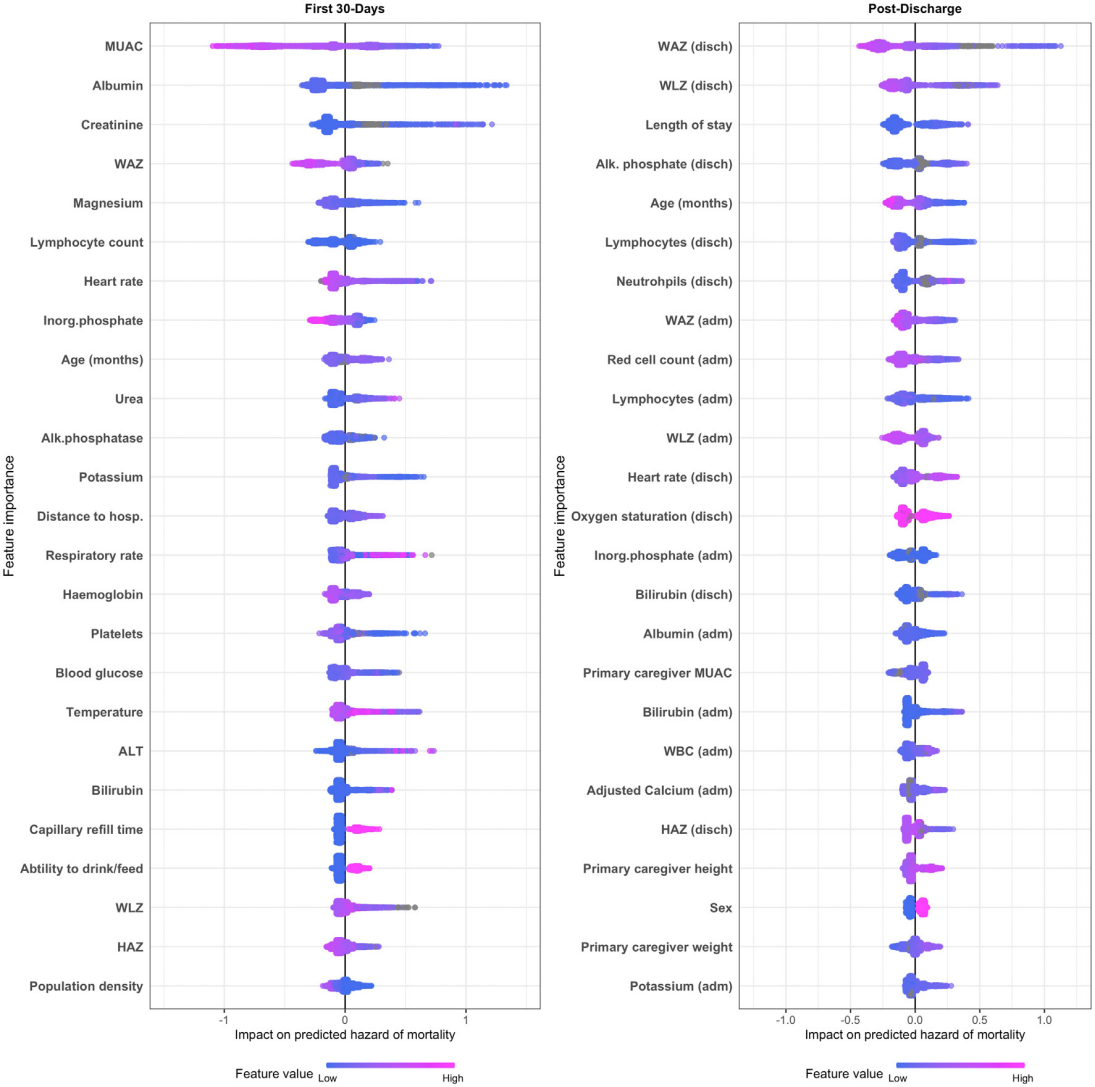
**Top 25 predictors of 30-day and 180-day mortality.** The 25 variables most influential variables in each model are display in descending order. Each participant has one dot on each variable line, this dot is colored by the value of that variable–pink for a high value, blue for a low value, grey for a missing value. The dots are positioned along the x-axis according to contribution of that variable to the child's predicted risk, left-side indicating the variable lower the predicted risk and the right-side increased the risk. For example, the pink dots on the left side of the MUAC variable in the 30-day model indicate that high MUAC was associated with lower risk of death. adm—admission, Alk. phosphatase—Alkaline Phosphatase, ALT—Alanine transaminase, disch.—discharge, HAZ—height-for-age z-score, hosp. —hospital, ICU—intensive care unit admission, Inorg. phosphate—inorganic phosphate, MUAC—mid upper arm circumference, WAZ—weight-for-age z-score, WLZ—weight-for-height z-score.

There were 234 (7.5%) deaths in the 30-days following admission. The median time-to-death was 5 days (IQR: 1, 11 days). The median C-statistic (AUC) of the 30-days models was 0.80 (IQR: 0.76, 0.83). The 25 variables providing the most information to the model are displayed in Fig. 2. MUAC was the strongest predictor of death. Twelve of the most influential 25 predictors were biochemical or haematological parameters. All four anthropometric measures (MUAC, WHZ, WAZ, LAZ) as well as younger patient age, lower heart rate, higher respiratory rate, slower capillary refill time, lower temperature, inability to drink or feed, far distance to the nearest hospital, and residing in a low population density area were in the most influential 25 predictors of death. Two clinical diagnoses/syndromes were among the most influential 50 predictors of mortality (Appendix 3, Fig. S1); clinician diagnosed sepsis increased the predicted risk of death while caregiver reported diarrhoea reduced the predicted risk. Some clinical syndromes are included in the description of 30-day mortality clusters below.

**Fig. 3:**
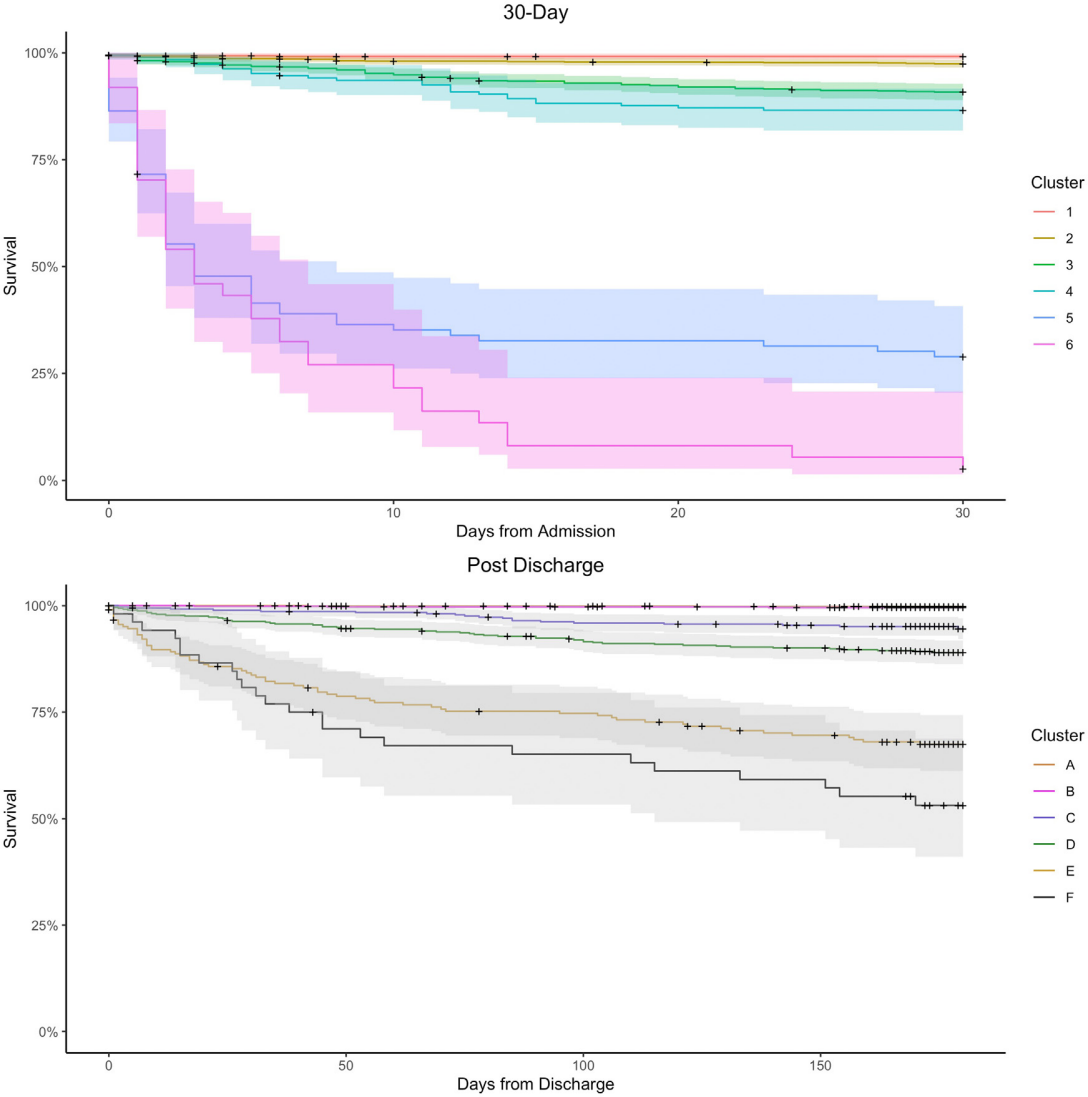
**Kaplan Meier survival curves for the clusters derived from the 30-day and post-discharge models.** Children at risk and number events in each cluster are given for major timepoints in Appendix 4, Supplementary Table S3.

**Fig. 4:**
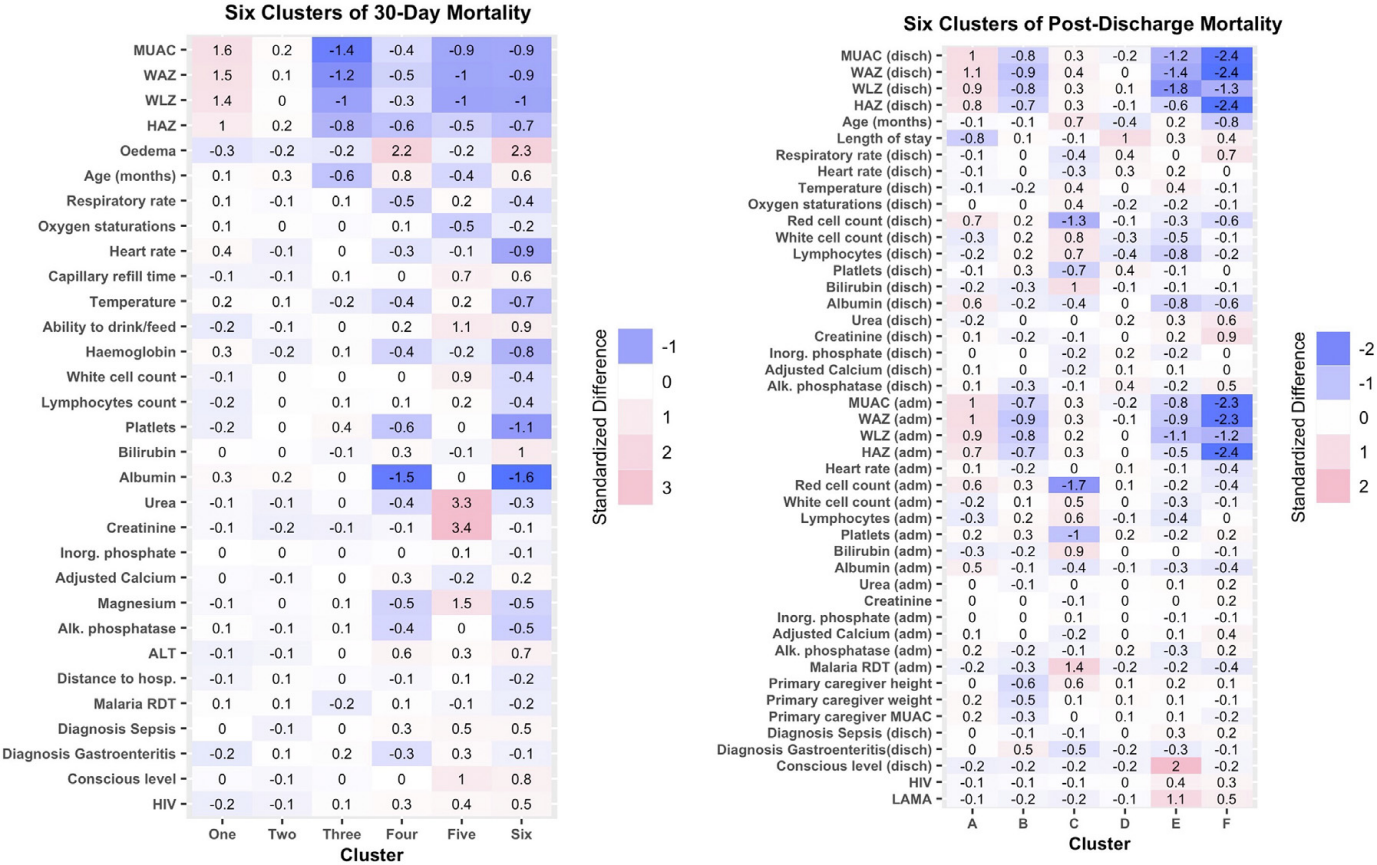
**Phenotypes of 30-dayand post-dischargemortality.** The color and value of each cell represents the standardised difference between the mean of the cluster and the total sample mean for the relevant variable, i.e. (Mean_cluster_–Mean_sample_)/Standard Deviation_sample_. Alk. phosphatase—Alkaline Phosphatase, ALT—Alanine transaminase, disch—discharge, HAZ—height-for-age z-score, HIV- human immunodeficiency virus, hosp.—hospital, Inorg. phosphate—inorganic phosphate, LAMA—leaving against medical advice, MUAC—mid upper arm circumference, RDT—rapid diagnostic test, WAZ—weight-for-age z-score, WLZ—weight-for-height z-score.

Among the six 30-day mortality clusters (Table 2, Fig. 3), 1915 children (61%) fell within the two lowest mortality clusters. Cluster one included 727 children (23%) and six deaths (1% cumulative mortality) with a median of 0 days between enrolment and death (IQR 0–0 days). This lowest mortality cluster had a mean MUAC that was 1.6 SD higher than the mean of the other clusters (Fig. 4). Similarly, WAZ (1.5 SD), HAZ (1.0 SD), and WHZ (1.4 SD) were higher in cluster one than the other clusters. Cluster two included 1188 children (38%), and thirty deaths (3% cumulative mortality) with a median time to death of four days (IQR: 1–9). This second lowest mortality cluster had no features that were greater than 0.5 SD from the mean of other clusters.

**Table 2:** The size and outcomes of 30-day mortality clusters, and the post-discharge mortality clusters.

30-day mortality						
	Cluster 1	Cluster 2	Cluster 3	Cluster 4	Cluster 5	Cluster 6
**N**	727	1188	880	187	81	37
**Died**	6	30	80	25	57	36
**Mortality (95% CI)**^a^	0.01 (0, 0.02)	0.03 (0.02, 0.04)	0.09 (0.07, 0.11)	0.13 (0.09, 0.19)	0.71 (0.62, 0.82)	0.97 (0.92, 1)
**Days to death, median (IQR)**	0 (0, 0)	4 (1, 8.75)	9 (3, 16)	11 (4, 14)	2 (1, 5)	3 (1, 7.75)


**Post-discharge mortality**						
	Cluster A	Cluster B	Cluster C	Cluster D	Cluster E	Cluster F
**N**	1043	710	373	493	215	52
**Died**	2	3	19	53	65	24
**Mortality (95% CI)**a	0.002 (0.00, 0.01)	0.004 (0.00, 0.01)	0.05 (0.03, 0.08)	0.11 (0.08, 0.14)	0.3 (0.25, 0.37)	0.46 (0.34, 0.62)
**Days to death, median (IQR)**	83 (62.5, 103.5)	46 (32, 92.5)	78 (42, 94.5)	56 (23, 100)	30 (8, 70)	35.5 (18, 91.3)

IQR—interquartile range.

a Kaplan Meier estimated cumulative mortality proportion were 1.0 would indicated 100% mortality in the observed period, and 0.5 would indicate 50% mortality.

Cluster three included 880 (28%) children and 80 deaths within 30-days (9%). This cluster was characterised by lower MUAC (−1.4 SD) and age (−0.6 SD) compared to the other clusters. Cluster four included 187 (6%) children, of whom 25 died (13% cumulative mortality). This cluster selected children with nutritional oedema (2.2 SD) and lower albumin (−1.5 SD), but without many of the features associated with mortality in the highest risk clusters, such as consciousness level, oral intake, lymphocyte count or signs of sepsis. Clusters 3 and 4 were associated with later mortality than the other clusters, with a median time to death of nine (IQR: 3–6) and eleven (IQR: 4–14) days, respectively.

Clusters five and six had extremely high mortality rates and collectively included 118 (4%) of the recruited children. Cluster five included 81 children (3%), of whom 57 (70% cumulative) died. Cluster six included 37 children (1%) and 36 deaths (97% cumulative mortality). These extreme risk clusters had median days to death of 2 (IQR: 1–5) and 3 (IQR: 1–7.8) respectively. Children in cluster five were characterised by high serum urea (3.3 SD) and creatinine (3.4 SD). Cluster six was characterised by nutritional oedema (2.3 SD) with a depressed platelet count (−1.1 SD), lower consciousness level (0.8 SD), reduced oral intake (0.9 SD) and increased capillary refill time (0.6 SD). Overall clusters four, five and six included 10% of the recruited children and half (50%) of all deaths observed within 30 days.

Sensitivity analyses in which the clustering algorithm returned four, five, seven and eight clusters are detailed in Appendix 3, Fig. S2. The composition of the 30-day mortality clusters identified using six clusters was remarkably similar across these analyses, with the two lowest risk clusters remaining largely unchanged. Similarly, a cluster defined by higher serum urea and creatinine and one or more clusters of children with nutritional oedema and lower serum albumin were shared across the sensitivity analyses.

### Post-discharge mortality

In the 6-months following hospital discharge, 2874 children were discharged alive and 166 (6%) children died. The median time to death was 45 days (IQR of 15–98 days). The median C-statistic (AUC) of the post-discharge XGBoost models was 0.74 (IQR: 0.68, 0.78). The 25 most informative variables were dominated by anthropometric, haematological, and biochemical parameters (Fig. 2). Low WAZ was the strongest predictor of mortality. Increased length of stay was also an important predictor, as were younger age, male sex, and having a lower heart rate at discharge. Caregiver anthropometric measures were also in the most influential 25 predictors. No WHO/IMCI syndromes associated with the index hospitalisation were among the most influential predictors, but we do include WHO/IMCI syndromes in the description of post-discharge mortality clusters below.

Two low mortality clusters were identified (Table 2, Fig. 3) and collectively these clusters included 1753 (61%) children. Cluster A included 1043 children (36%) and two deaths (0.2% cumulative mortality) which occurred a median of 83 days (IQR: 63, 104) after discharge. Cluster B included 710 (25%) children with three deaths (0.04% cumulative mortality), and a median time between discharge and death of 46 days (IQR: 32, 94). Cluster A was characterised by higher anthropometric measures in comparison to other clusters (Appendix 3, Fig. S6), including WAZ (1.1 SD) and WLZ (0.9 SD) at discharge, shorter length of stay (−0.8 SD), higher red blood cell counts (0.7 SD) and higher albumin (0.6 SD) than the other clusters. Children in Cluster B had lower anthropometry at discharge, WAZ (0.9 SD) and WLZ (−0.8), in comparison to the other clusters, and a higher probability of a gastroenteritis diagnosis (0.5 SD).

Cluster C included 373 (13%) children with 19 deaths (5% cumulative mortality), and a median of 78 (IQR: 42–95) days between discharge and death. This cluster had a higher probability of a positive malaria test (1.4 SD), lower red cell count (−1.3 SD), higher bilirubin (1.0 SD), raised white cell count (0.8 SD) and lower platelet count (−0.7 SD) than other clusters. Cluster D included 493 (17%) children, of whom 53 (11% cumulative mortality) died. The median days-to-death in this cluster was 56 (IQR: 23, 100). This cluster was defined by a longer length of stay in hospital (1.1 SD) but without clear differences in anthropometric status or clinical signs at admission or discharge.

Clusters E and F included 267 (9%) of the children and had extremely high mortality rates with 65 (30% cumulative mortality) and 24 (46% cumulative mortality) deaths respectively. Cluster E contained 215 (7%) children, with a median of 30 (IQR 8, 70) days to death. Children in this cluster more often left against medical advice (1.1 SD) and had a reduced consciousness level at discharge (2.0 SD). Cluster E was also defined by lower anthropometry at discharge (−1.2 SD MUAC, −1.8 SD WLZ, −1.4 SD WAZ, −0.6 SD HAZ). Cluster F included 52 (2%) children with a median of 36 (IQR 18, 91) days to death. These children were characterised by very low anthropometry (−2.4 SD MUAC, −1.3 SD WLZ, −2.4 SD WAZ, −2.4 SD HAZ) and signs of illness at discharge, including higher respiratory rates (0.7 SD), higher creatinine (0.9 SD), and higher urea 0.6 (SD). Children in cluster F were also younger than other clusters (−0.8 SD).

Sensitivity analyses varying the number of post-discharge clusters were less consistent than the 30-day model (Appendix 3, Fig. S7). Most of these sensitivity analyses included one or more low risk clusters of children with higher anthropometry and shorter hospital stay, and at least one high risk cluster of children with wasting and either reduced consciousness or high creatinine. A cluster defined by lower red blood cells and positive malaria tests was identified in six, seven, and eight cluster variations. The eight cluster analysis split cluster D, defined by longer hospital stay without a clear anthropometric or clinical pattern, into three different clusters, defined by children with long stays, a low lymphocyte counts, and a third by raised alkaline phosphatase.

### Simplification and models’ performance

We repeated the train and test pipeline with a decreasing number of variables, beginning with the 50 most informative predictors and iteratively dropping the least informative variables (Appendix 3, Fig. S9a). In the 30-day dataset, there were modest declines in C-statistic between models using 50 variables and those using 10 variables. Fewer than 10 variables substantially decreased 30-day performance. The post-discharge mortality mean C-statistic was maintained between 0.73 and 0.80 in models including 8–50 variables, but model performance became substantially weaker with less than eight variables (Appendix 3, Fig. S9b).

## Discussion

Risk phenotypes were identified among children hospitalised with acute illnesses under two years of age by a discrete set of anthropometric, clinical and laboratory features. These machine-generated clusters suggest that a large proportion of children were at low risk of both 30-day and post-discharge mortality, while much smaller clusters of children were at very high risk of death. Echoing our investigator-driven analysis,^5^ these models suggest that syndrome-agnostic risk stratification at admission and discharge could be used to re-distribute resources toward high-risk groups. Among lower risk children, risk stratification may limit the use of unnecessary interventions thus reducing nosocomial infection,^19^ antimicrobial resistance, and catastrophic household medical expenses.^20^

Both models selected laboratory variables to be among the best predictors of mortality, including red blood cell, platelet and lymphocyte counts, in addition to creatinine, urea, and albumin levels. Risk-based management algorithms would benefit from inclusion of these parameters, but consistent provision of clinical laboratory tests are limited by logistic and financial barriers.^21^^,^^22^ Uptake of these simple laboratory tests into current guidelines is hampered by stock outs, poor quality control, and barriers to the timely return of result to the treating clinicians. However, large investments in new technologies and laboratory services for HIV, malaria and tuberculosis have helped 84% of people living with HIV to know their status,^23^ revolutionised management guidelines for fever,^24^ and facilitated a 20% increase in testing for drug resistance among patients with tuberculosis between 2017 and 2019.^25^ Similar investments in routine laboratory testing could have profound impact on the inpatient and post-discharge management of paediatric illness.

Anthropometric indices are important prognostic measures,^26^^,^^27^ and causal models built using this dataset show that anthropometry captures a broad range of pathways to mortality.^5^ Children with nutritional oedema were identified by our model as a unique mortality cluster. Nutritional oedema, or kwashiorkor, is a condition characterised by bi-pedal oedema and is associated childhood wasting, but with an unclear aetiology. Children with nutritional oedema have discernibly different protein, lipid and glucose metabolism in comparison to children with wasting.^28^ These children also have a reduced capacity to handle oxidative stress, and specific faecal microbiome changes.^28^ Collectively, these biological differences lend credibility to our observation that nutritional oedema should be considered a unique risk phenotype.

Specific WHO/IMCI clinical syndromes, were not important predictors of risk in our models. All enrolled children were acutely unwell, and these syndromes would be associated with mortality if they were compared to healthy children. Children included in this study were managed at referral facilities where uncomplicated diarrhoea, malaria and pneumonia are managed very effectively.^29^ The majority of deaths occurred among children with severe complications (e.g., renal insufficiency) or comorbidities (e.g., wasting). Incremental changes to WHO/IMCI syndromic guidelines may have a limited impact on inpatient and post-discharge mortality, and larger reductions may require a focus on the identification and management of cases with complications or comorbidities.

In predicative modelling, the simplest model that approximates the best predictive performance is usually preferred.^6^ We developed a complex model, with access to hundreds of variables, but found models with 8–10 variables largely replicated predicative performance. Our models were only modestly better than previous developed algorithms using simpler techniques.^2^^,^^11^^,^^27^^,^^30^ This suggests that the small increase in predictive performance that a bedside artificial intelligence application might yield over a simple clinical algorithm may not justify the cost of implementing such a system. It is unlikely that there is an undiscovered combination of clinical signs and symptoms at admission or discharge that will predict mortality with extremely high accuracy. Developing algorithms based on changes in clinical status over fixed time periods, e.g., response to 24- or 48-h of treatment, may be more useful to clinicians than attempts to rearrange signs as a single timepoint.^30^

The CHAIN cohort was a multi-country study that achieved harmonised and systematic data collection with very high retention among a large and heterogenous population of children. However, our analysis also has limitations. We cannot make causal inference and any observed associations should be tested in external datasets using a causal inference framework where confounders such as site can be included in the model. Variables in our models are conditional on all the other variables within the model. For example, a pneumonia diagnosis is not highly ranked in our model because mediators, such as low pulse oximetry and high heart rate, were also in the model. We were not able to include variables related to socioeconomic status of children in the 30-day model, this limits the 30-day model to a biomedical view of acute mortality and omits the social context of those deaths. There are laboratory tests, or other clinical variables, that may be more informative than those available in this dataset, such as C-reactive protein. These data were also collected at research facilities with active monitoring of syndrome specific guideline adherence, which may limit generalisability, and may explain the weak associations between syndromes and mortality. Finally, despite achieving low lost-to-follow-up, it is still possible that the unknown outcomes of these children could bias the result, if they were disproportionately more likely to die.

In conclusion, novel clusters of children with acute illnesses at both high and low risk of mortality were defined by cross-cutting risk factors, including lower anthropometry, low red cell counts, low white cell counts, high platelet counts, hypoalbuminemia, and biochemical markers of renal insufficiency. WHO/IMCI syndromes did not substantially contribute to risk prediction, suggesting that severity of illness, rather than cause, is more important in predicting outcome. However, these complex models only offered modest improvements in accuracy compared simpler tools using a limited subset of variables. This suggests that investing in artificial intelligence algorithms to improve LMIC paediatric management may prove less effective than expanding access to reliable bedside or laboratory assessment. Incorporating these laboratory measures into clinical guidelines, may help target life-saving resources at children at highest risk of death which may lower the mortality during and after acute childhood illnesses.

## Contributors

Funding: JAB, JLW. Design: SF, KDT, JH, AFS, CLL, DMD, EMu, JAB, JLW, JJ, MMa, MJC, MMN, PS, RHB, SMo, SAA, TA, WPV. Supervision: AHD, AFS, BOS, CB, CLL, CMan, CJM, DMD, EMb, EMu, GSM, JAB, JM, JT, JLW, KDT, MMa, MJC, MT, PS, RHB, SMo, SMw, SAA, TA, WPV, ZK. Data collection: AMS, AFK, COO, CMan, CJM, CL, DA, DM, EMb, IO, JN, JM, LS, MH, MT, PPH, PS, SMw, SNS, SMA, SB. Data management: AHD, BSG, CAO, CB, CMar, EC, JDC, JJ, MMb, MMN, NN, PA, RMB. Laboratory analysis: AHD, CT, RMB, ZK. Coordination: AHD, AFS, BOS, CT, CAO, CB, COO, CLL, CMar, CL, DIM, DC, JAB, JN, JM, JT, JLW, KDT, MJC, MT, MMb, NN, PA, PS, RHB, RMB, SMo, WPV, ZK. Analysis: SF, KDT, GG, NN. Interpretation: AHD, BSG, CAO, CB, CLL, DB, DMD, EMu, IO, JAB, JLW, KDT, MJC, MMN, PS, RHB, RMB, SMo, SAA, TA, WPV, SF, JH. First draft: KDT, SF, JLW, JAB. Critical review: AHD, AMS, AFK, AFS, BOS, BSG, CT, CAO, CB, COO, CLL, CMan, CJM, CMar, CL, DB, DA, DM, DMD, DIM, DC, EMb, EMu, GSM, IO, JAB, JN, JM, JT, JDC, JLW, JJ, KDT, LS, MMa, MH, MJC, MT, MMb.

KDT, NN, GG and SF all access to the data and verified the results presented.

## Data sharing statement

The CHAIN cohort data and analysis code are deposited and may be requested at: https://dataverse.harvard.edu/dataset.xhtml?persistentId=doi:10.7910/DVN/5H5X0P.

## Declaration of interests

All authors declare no competing interests.

## Appendix A Supplementary data

Supplements

CHAIN_Co-Author_PubMed_Name_List

## References

[1] UNICEF (2017).

[2] Nemetchek B.R., Liang L.D., Kissoon N. (2018). Predictor variables for post-discharge mortality modelling in infants: a protocol development project. Afr Health Sci.

[3] Hossain M., Chisti M.J., Hossain M.I., Mahfuz M., Islam M.M., Ahmed T. (2017). Efficacy of World Health Organization guideline in facility-based reduction of mortality in severely malnourished children from low and middle income countries: a systematic review and meta-analysis. J Paediatr Child Health.

[4] (2019). Childhood Acute Illness and Nutrition (CHAIN) Network: a protocol for a multi-site prospective cohort study to identify modifiable risk factors for mortality among acutely ill children in Africa and Asia. BMJ Open.

[5] Diallo A.H., Sayeem Bin Shahid A.S.M., Khan A.F. (2022). Childhood mortality during and after acute illness in Africa and south Asia: a prospective cohort study. Lancet Glob Health.

[6] Kuhn M., Johnson K. (2013).

[7] Moncada-Torres A., van Maaren M.C., Hendriks M.P., Siesling S., Geleijnse G. (2021). Explainable machine learning can outperform Cox regression predictions and provide insights in breast cancer survival. Sci Rep.

[8] Hu C.-A., Chen C.-M., Fang Y.-C. (2020). Using a machine learning approach to predict mortality in critically ill influenza patients: a cross-sectional retrospective multicentre study in Taiwan. BMJ Open.

[9] Thorsen-Meyer H.-C., Nielsen A.B., Nielsen A.P. (2020). Dynamic and explainable machine learning prediction of mortality in patients in the intensive care unit: a retrospective study of high-frequency data in electronic patient records. Lancet Digit Health.

[10] Castaldi P.J., Boueiz A., Yun J. (2020). Machine learning characterization of COPD subtypes: insights from the COPDGene study. Chest.

[11] van den Brink D.A., de Meij T., Brals D. (2020). Prediction of mortality in severe acute malnutrition in hospitalized children by faecal volatile organic compound analysis: proof of concept. Sci Rep.

[12] Ginsburg A.S., Delarosa J., Brunette W. (2015). mPneumonia: development of an innovative mHealth application for diagnosing and treating childhood pneumonia and other childhood illnesses in low-resource settings. PLoS One.

[13] Shah R., Jenda G., Lwesha V., Nsona H., Dadlani P., Swedberg E. (2018). An integrated diagnostic device for neonatal sepsis and childhood pneumonia. J Public Health Afr.

[14] The humanitarian data exchange: health facility location data for sub-Saharan Africa, Bangladesh and Pakistan. https://data.humdata.org/dataset.

[15] XgBoost package. https://github.com/dmlc/xgboost.

[16] Chen T., Guestrin C. (2016).

[17] Lundberg S., Lee S. (2017).

[18] Pedregosa F., Varoquaux G., Gramfort A. (2011). Scikit-learn: machine learning in {P}ython. J Mach Learn Res.

[19] Richards M.J., Edwards J.R., Culver D.H., Gaynes R.P. (1999). Nosocomial infections in pediatric intensive care units in the United States. National nosocomial infections surveillance system. Pediatrics.

[20] Sheikh N., Sarker A.R., Sultana M. (2022). Disease-specific distress healthcare financing and catastrophic out-of-pocket expenditure for hospitalization in Bangladesh. Int J Equity Health.

[21] Wilson M.L., Fleming K.A., Kuti M.A., Looi L.M., Lago N., Ru K. (2018). Access to pathology and laboratory medicine services: a crucial gap. Lancet Lond Engl.

[22] Horton S., Sullivan R., Flanigan J. (2018). Delivering modern, high-quality, affordable pathology and laboratory medicine to low-income and middle-income countries: a call to action. Lancet Lond Engl.

[23] UNAIDS Global HIV & AIDS statistics - fact sheet. https://www.unaids.org/en/resources/fact-sheet.

[24] Means A.R., Weaver M.R., Burnett S.M., Mbonye M.K., Naikoba S., McClelland R.S. (2014). Correlates of inappropriate prescribing of antibiotics to patients with malaria in Uganda. PLoS One.

[25] WHO (2020).

[26] Mwangome M.K., Fegan G., Fulford T., Prentice A.M., Berkley J.A. (2012). Mid-upper arm circumference at age of routine infant vaccination to identify infants at elevated risk of death: a retrospective cohort study in the Gambia. Bull World Health Organ.

[27] Ngari M.M., Fegan G., Mwangome M.K. (2017). Mortality after inpatient treatment for severe pneumonia in children: a cohort study. Paediatr Perinat Epidemiol.

[28] Bhutta Z.A., Berkley J.A., Bandsma R.H.J., Kerac M., Trehan I., Briend A. (2017). Severe childhood malnutrition. Nat Rev Dis Primers.

[29] Tickell K.D., Mangale D.I., Tornberg-Belanger S.N. (2019). A mixed method multi-country assessment of barriers to implementing pediatric inpatient care guidelines. PLoS One.

[30] Wen B., Brals D., Bourdon C. (2021). Predicting the risk of mortality during hospitalization in sick severely malnourished children using daily evaluation of key clinical warning signs. BMC Med.

