# Supplementary material for: Characterising paediatric mortality during and after acute illness in Sub-Saharan Africa and South Asia: a secondary analysis of the CHAIN cohort using a machine learning approach
Source: eClinicalMedicine. 2023 Feb 6;57:101838. doi: 10.1016/j.eclinm.2023.101838 (PMC9941052; doi:10.1016/j.eclinm.2023.101838)
Supplement: Supplements [file mmc1.docx]

**APPENDIX 1: Methodological Appendix**

Rationale for parent study including and exclusion criteria: The parent study was focused on identifying mechanism of mortality among acutely ill children, and not those with traumatic injury. The collected investigators of the parent study believed that the mechanisms of death among children with traumatic injury are sufficiently different from those with acute illnesses that they should be excluded from the study. We have added this information into a methodological appendix.

30-day mortality analysis: We believe 30-day mortality to be a statistically better outcome than inpatient mortality, as the duration of the inpatient stay is conditional on the child’s clinical recovery and is therefore highly subject to informative censoring.

Additionally, we believe the goal of inpatient care goes beyond promoting survival to discharge, and extends to ensuring the child is able to return to the home environment without risk of mortality. Given the high post-discharge mortality occurring in the days following discharge from hospital in many LMIC settings, we cannot assume that a decision to discharge a child is indicative of a safe return to home-care. Therefore, we believe 30-day mortality more accurately reflects the success of inpatient management.

Finally, we noted that 30-day mortality analyses are increasingly common in the acute illness literature.

Considerations in supervised vs unsupervised approaches: We aimed to describe phenotypes associated with mortality and survival based on the clinical, anthropometric, laboratory, and sociodemographic features. To achieve this goal we implemented a “explainable machine learning” approach, beginning with a supervise model that predicted mortality. A commonly implemented alternative approach is to begin with an unsupervised technique to reduce clinical, anthropometric, laboratory, and sociodemographic to smaller set of modules summarizing variability across participants before including these modules into survival models. However, there are some important challenges in the unsupervised approach. Unsupervised approaches collapse information without reference to the outcome. This means they may discard small areas of heterogeneity in the data that may be important to predicting the outcome. As Kuhn & Johnson stated (Applied Predictive Modelling, Springer 2016), “If the predictive relationship between the predictors and the response is not connected to the predictor’s variability then the derived principle components will not provide a suitable relationship with the response. In this case a supervised approach, like PLS will derive components while simultaneously considering the corresponding response”. While this statement is about principal component analysis, we believe it is applicable to all unsupervised techniques. These approaches also tend to favor highly heterogeneous continuous variables over binary variables, even if the former is entirely unrelated to the outcome. As a clinical dataset, we had many variables with relatively narrow ranges (i.e. those highly conserved in biological systems, like potassium) or binary distributions, that are potentially very important to mortality. Finally, the composite modules derived from an unsupervised model can be hard to interpret, as they borrow information from many different correlated variables. Interpretating these composite modules can become very subjective.

In addition to avoiding some of the limitations of an unsupervised approach, the current analysis has several other advantages. The Xgboost model is able to capture non-linearity in relationships, and it also allows us to rank order the relative importance of each individual variable in mortality prediction within our dataset. An unsupervised approach would not offer insights into the importance of individual variables within individuals, nor would we have been able to test the effect of iteratively removing each variable on predictive performance (see appendix Figure 9a). Finally, the SHAP values and the clustering algorithm also allows us to assess the number of children that would fall into each, which allows us to compute the relative proportion of mortality that was covered by each cluster. Our approach also has limitations, foremost amongst them are the challenges of choosing the number of clusters and the potential for subjectivity in the interpretation of the clusters. To address these concerns, we have given detailed information about alternative cluster numbers, and the composition of each cluster.

Extreme Gradient Boosting: Extreme Gradient Boosting is a flexible ensemble approach, which is able to model non-linear methods, include missing data as a potential predictor, and penalize models to avoid a risk of overfitting. Extreme Gradient Boosting has proven to be one of the effective predicative modelling tools across a wide variety of data challenges. For additional information see: Chen T, Guestrin C. XGBoost: A Scalable Tree Boosting System. In 2016.

Spectral clustering: Algorithm groups observations into clusters. Statistical simulation experiments have shown spectral clustering to be among the optimal methods of cluster identification currently available. For futher information reviewhttps://scikit-learn.org/stable/auto_examples/cluster/plot_cluster_comparison.html#sphx-glr-auto-examples-cluster-plot-cluster-comparison-py, and  [Scikit-learn: Machine Learning in Python](http://jmlr.csail.mit.edu/papers/v12/pedregosa11a.html), Pedregosa *et al.*, JMLR 12, pp. 2825-2830, 2011.

Additional variable definitions

Syndromic diagnoses – Diagnoses such as pneumonia, diarrhea, malaria, meningitis were included in this analysis but were diagnosed by the treating clinicians, and were not computed during the data analysis using available clinical information. Clinicians were encouraged to follow the WHO recommendations/definitions of major paediatric conditions. However, severity of illness (severity of dehydration, pneumonia severity) were by the data analysis team using the relevant WHO criteria.

Leaving against medical advice – A family/child that either absconds from the ward or leaves against medical advice because they were not judged medically fit to return home.

Length of stay – Numbers of days between admission and discharge.

**VARIABLES INCLUDED IN 30-DAY RISK PREDICITION DATASET**

| **CLINICAL** | |  |
| --- | --- | --- |
| complaints of fever at admission | |  |
| complaints of vomiting at admission | |  |
| complaints of lethargy at admission | |  |
| complaint of difficultbreath at admission | |  |
| complaint of diarr_under14days at admission | |  |
| complaint of convulsions at admission | |  |
| complaint of cough_under14days at admission | |  |
| complaint of diarr_over14days at admission | |  |
| complaint of alt_consciousness at admission | |  |
| complaint of cough_14days at admission | |  |
| complaint of blood_stool at admission | |  |
| complaint of not_feeding at admission | |  |
| complaint of skin_lesion at admission | |  |
| complaint of poor_feeding at admission | |  |
| complaint of development_delay at admission | |  |
| complaint of oedema at admission | |  |
| complaint of other at admission | |  |
| temperature at admission | |  |
| respiratory rate at admission | |  |
| heart rate at admission | |  |
| oxygen sataturations taken at admission | |  |
| oxygen saturation at admission | |  |
| medications in last 7 days: no at admission | |  |
| medications in last 7 days: antibiotic at admission | |  |
| medications in last 7 days: antimalaria at admission | |  |
| medications in last 7 days: traditional at admission | |  |
| medications in last 7 days: deworming at admission | |  |
| medications in last 7 days: vitamin at admission | |  |
| medications in last 7 days: yes_unknown at admission | |  |
| medications in last 7 days: other at admission | |  |
| urine volume at admission | | |
| airway check at admission | | |
| breathing: normal at admission | | |
| breathing: central_cyanosis at admission | | |
| breathing: nasal_flaring at admission |  |  |
| breathing: reduced_air_entry at admission |  |  |
| breathing: wheeze at admission |  |  |
| breathing: acidotic_breathing at admission |  |  |
| breathing: grunting at admission |  |  |
| breathing: indrawing at admission |  |  |
| breathing: crackles at admission |  |  |
| breathing: dull_percussion at admission |  |  |
| breathing: nodding at admission |  |  |
| capillary refill at admission |  |  |
| cold peripheries at admission |  |  |
| conscious level at admission |  |  |
| fontanelle at admission |  |  |
| tone at admission |  |  |
| posture at admission |  |  |
| lethargic at admission |  |  |
| sunken eyes at admission |  |  |
| skin pinch at admission |  |  |
| drink/feed at admission |  |  |
| abdominal exam: normal at admission |  |  |
| abdominal exam: distension at admission |  |  |
| abdominal exam: hepatomegaly at admission |  |  |
| abdominal exam: tenderness at admission |  |  |
| abdominal exam: splenomegaly at admission |  |  |
| abdominal exam: other at admission |  |  |
| rickets signs: none at admission |  |  |
| rickets signs: wristwidening at admission |  |  |
| rickets signs: rachiticrosary at admission |  |  |
| rickets signs: swollenknees at admission |  |  |
| rickets signs: bowlegs at admission |  |  |
| rickets signs: frontalbossing at admission |  |  |
| jaundice at admission |  |  |
| head & neck exam: mouth_normal at admission |  |  |
| head & neck exam: ears_normal at admission |  |  |
| head & neck exam: eyes_normal at admission |  |  |
| head & neck exam: oral_ulceration at admission |  |  |
| head & neck exam: pus_ear at admission |  |  |
| head & neck exam: conjunctivitis at admission |  |  |
| head & neck exam: oral_candidiasis at admission |  |  |
| head & neck exam: swelling_behind_ear at admission |  |  |
| head & neck exam: eye at dischargearge at admission |  |  |
| head & neck exam: stomatitis at admission |  |  |
| head & neck exam: lymphadenopathy at admission |  |  |
| head & neck exam: visual_impairment at admission |  |  |
| skin: normal at admission |  |  |
| skin: hyperpigmentation at admission |  |  |
| skin: depigmentaton at admission |  |  |
| skin : broken_skin at admission |  |  |
| skin: dermatitis at admission |  |  |
| skin: flaky_paint at admission |  |  |
| skin: cellulitis at admission |  |  |
| skin: impetigo at admission |  |  |
| skin: pustules at admission |  |  |
| skin: vesicles at admission |  |  |
| skin: desquamation at admission |  |  |
| skin: macular_papular at admission |  |  |
| rash site: trunk at admission |  |  |
| rash site: face_scalp at admission |  |  |
| rash site: legs at admission |  |  |
| rash site: palms_soles at admission |  |  |
| rash site: buttocks at admission |  |  |
| rash site: arms at admission |  |  |
| rash site: perineum at admission |  |  |
| rash site: NA at admission |  |  |
| outpatient program at admission |  |  |
| treated with food_supplement prior to admission |  |  |
| Any breastfeeding at admission |  |  |
| breastfeeding: exclusive at admission |  |  |
| Age breastfeeding stopped |  |  |
| weaning foods: sugar_water at admission |  |  |
| weaning foods: formula_powder_milk at admission |  |  |
| weaning foods: animal_milk at admission |  |  |
| weaning foods: fruit_juice at admission |  |  |
| weaning foods: tea at admission |  |  |
| weaning foods: other at admission |  |  |
| weaning foods: water at admission |  |  |
| weaning foods: porridge_pulp at admission |  |  |
| weaning foods: nothing at admission |  |  |
| weaning foods: pure_honey at admission |  |  |
| weaning foods: glycerine at admission |  |  |
| weaning foods: gutthi_gripe at admission |  |  |
| clinician mpression of risk at admission |  |  |
| malaria rdt at admission |  |  |
| blood glucose at admission |  |  |
| diagnosis: lrti_pneumonia at admission |  |  |
| diagnosis: bronchiolitis at admission |  |  |
| diagnosis: urti at admission |  |  |
| diagnosis: tb at admission |  |  |
| diagnosis: otitis at admission |  |  |
| diagnosis: asthma at admission |  |  |
| diagnosis: anaemia at admission |  |  |
| diagnosis: sicklecell at admission |  |  |
| diagnosis: thalassaemia at admission |  |  |
| diagnosis: renal at admission |  |  |
| diagnosis: nephrotic at admission |  |  |
| diagnosis: nephritis at admission |  |  |
| diagnosis: liver at admission |  |  |
| diagnosis: ileus at admission |  |  |
| diagnosis: cardiac at admission |  |  |
| diagnosis: gastroenteritis at admission |  |  |
| diagnosis: sepsis at admission |  |  |
| diagnosis: malaria at admission |  |  |
| diagnosis: soft_tissue at admission |  |  |
| diagnosis: uti at admission |  |  |
| diagnosis: hiv_related at admission |  |  |
| diagnosis: measles at admission |  |  |
| diagnosis: varicella at admission |  |  |
| diagnosis: osteomyelitis at admission |  |  |
| diagnosis: febrile at admission |  |  |
| diagnosis: enteric_fever at admission |  |  |
| diagnosis: epilepsy at admission |  |  |
| diagnosis: meningitis at admission |  |  |
| diagnosis: encephalopathy at admission |  |  |
| diagnosis: hydrocephalus at admission |  |  |
| diagnosis: developmentaldelay at admission |  |  |
| diagnosis: cerebral_palsy at admission |  |  |
| lumbar puncture at admission |  |  |
| fast breathing at admission |  |  |
| grunting at admission |  |  |
| hypoxia at admission |  |  |
| respiratory at admission |  |  |
| conscious at admission |  |  |
| dehydration at admission |  |  |
| dysentery at admission |  |  |
| circulat at admission |  |  |
| rickets at admission |  |  |
| fever at admission |  |  |
| diarrhoea at admission |  |  |
| bglucose at admission |  |  |
| neuro at admission |  |  |
| sickle cell disease at admission |  |  |
| thalassaemia at admission |  |  |
| visualprob at admission |  |  |
| weight loss prior at admission |  |  |
| knowntb at admission |  |  |
| cough >14days at admission |  |  |
| known bcontact at admission |  |  |
| suspect tb at admission |  |  |
| on ART at admission |  |  |
| on ARVS at admission |  |  |
| hiv exposed |  |  |
| hiv status at admission |  |  |
| oedema at admission |  |  |
| MUAC at admisison |  |  |
| head circumference at admission |  |  |
| haz at admission |  |  |
| waz at admission |  |  |
| wlz at admission |  |  |
| DEMOGRAPHIC |  |  |
| sex at admission |  |  |
| african site |  |  |
| site |  |  |
| age in months at admission |  |  |
| population density near home |  |  |
| distance to study hospital |  |  |
| distance to nearest hospital to home |  |  |
| LABORATORY |  |  |
| sodium at admission |  |  |
| potassium at admission |  |  |
| calcium at admission |  |  |
| albumin at admission |  |  |
| alt at admission |  |  |
| alk_phosphate at admission |  |  |
| urea at admission |  |  |
| creatinine at admission |  |  |
| bilirubin at admission |  |  |
| inorganic phosphate at admission |  |  |
| magnesium at admission |  |  |
| red blood cells at admission |  |  |
| white blood cells at admission |  |  |
| platelets at admission |  |  |
| lymphocytes at admission |  |  |
| haemoglobin (altitude adjusted) at admission |  |  |

**APPENDIX 1.2: VARIABLES INCLUDED IN POST-DISCHARGE RISK PREDICITION DATASET**

| **CLINICAL** |
| --- |
| complaints of fever at admission |
| complaints of vomiting at admission |
| complaints of lethargy at admission |
| complaint of difficultbreath at admission |
| complaint of diarr_under14days at admission |
| complaint of convulsions at admission |
| complaint of cough_under14days at admission |
| complaint of diarr_over14days at admission |
| complaint of alt_consciousness at admission |
| complaint of cough_14days at admission |
| complaint of blood_stool at admission |
| complaint of not_feeding at admission |
| complaint of skin_lesion at admission |
| complaint of poor_feeding at admission |
| complaint of development_delay at admission |
| complaint of oedema at admission |
| complaint of other at admission |
| temperature at admission |
| respiratory rate at admission |
| heart rate at admission |
| oxygen sataturations taken at admission |
| oxygen saturation at admission |
| previous hospitalization at admission |
| medications in last 7 days: no at admission |
| medications in last 7 days: antibiotic at admission |
| medications in last 7 days: antimalaria at admission |
| medications in last 7 days: traditional at admission |
| medications in last 7 days: deworming at admission |
| medications in last 7 days: vitamin at admission |
| medications in last 7 days: yes_unknown at admission |
| medications in last 7 days: other at admission |
| urine volume at admission |
| airway check at admission |
| breathing: normal at admission |
| breathing: central_cyanosis at admission |
| breathing: nasal_flaring at admission |
| breathing: reduced_air_entry at admission |
| breathing: wheeze at admission |
| breathing: acidotic_breathing at admission |
| breathing: grunting at admission |
| breathing: indrawing at admission |
| breathing: crackles at admission |
| breathing: dull_percussion at admission |
| breathing: nodding at admission |
| capillary refill at admission |
| cold peripheries at admission |
| conscious level at admission |
| fontanelle at admission |
| tone at admission |
| posture at admission |
| lethargic at admission |
| sunken eyes at admission |
| skin pinch at admission |
| drink/feed at admission |
| abdominal exam: normal at admission |
| abdominal exam: distension at admission |
| abdominal exam: hepatomegaly at admission |
| abdominal exam: tenderness at admission |
| abdominal exam: splenomegaly at admission |
| abdominal exam: other at admission |
| rickets signs: none at admission |
| rickets signs: wristwidening at admission |
| rickets signs: rachiticrosary at admission |
| rickets signs: swollenknees at admission |
| rickets signs: bowlegs at admission |
| rickets signs: frontalbossing at admission |
| jaundice at admission |
| head & neck exam: mouth_normal at admission |
| head & neck exam: ears_normal at admission |
| head & neck exam: eyes_normal at admission |
| head & neck exam: oral_ulceration at admission |
| head & neck exam: pus_ear at admission |
| head & neck exam: conjunctivitis at admission |
| head & neck exam: oral_candidiasis at admission |
| head & neck exam: swelling_behind_ear at admission |
| head & neck exam: eye at dischargearge at admission |
| head & neck exam: stomatitis at admission |
| head & neck exam: lymphadenopathy at admission |
| head & neck exam: visual_impairment at admission |
| skin: normal at admission |
| skin: hyperpigmentation at admission |
| skin: depigmentaton at admission |
| skin : broken_skin at admission |
| skin: dermatitis at admission |
| skin: flaky_paint at admission |
| skin: cellulitis at admission |
| skin: impetigo at admission |
| skin: pustules at admission |
| skin: vesicles at admission |
| skin: desquamation at admission |
| skin: macular_papular at admission |
| rash site: trunk at admission |
| rash site: face_scalp at admission |
| rash site: legs at admission |
| rash site: palms_soles at admission |
| rash site: buttocks at admission |
| rash site: arms at admission |
| rash site: perineum at admission |
| rash site: NA at admission |
| outpatient program at admission |
| treated with food_supplement prior to admission |
| Any breastfeeding at admission |
| breastfeeding: exclusive at admission |
| Age breastfeeding stopped |
| weaning foods: sugar_water at admission |
| weaning foods: formula_powder_milk at admission |
| weaning foods: animal_milk at admission |
| weaning foods: fruit_juice at admission |
| weaning foods: tea at admission |
| weaning foods: other at admission |
| weaning foods: water at admission |
| weaning foods: porridge_pulp at admission |
| weaning foods: nothing at admission |
| weaning foods: pure_honey at admission |
| weaning foods: glycerine at admission |
| weaning foods: gutthi_gripe at admission |
| clinician mpression of risk at admission |
| malaria rdt at admission |
| blood glucose at admission |
| admitted ward/ICU at admission |
| diagnosis: lrti_pneumonia at admission |
| diagnosis: bronchiolitis at admission |
| diagnosis: urti at admission |
| diagnosis: tb at admission |
| diagnosis: otitis at admission |
| diagnosis: asthma at admission |
| diagnosis: anaemia at admission |
| diagnosis: sicklecell at admission |
| diagnosis: thalassaemia at admission |
| diagnosis: renal at admission |
| diagnosis: nephrotic at admission |
| diagnosis: nephritis at admission |
| diagnosis: liver at admission |
| diagnosis: ileus at admission |
| diagnosis: cardiac at admission |
| diagnosis: gastroenteritis at admission |
| diagnosis: sepsis at admission |
| diagnosis: malaria at admission |
| diagnosis: tb at admission |
| diagnosis: soft_tissue at admission |
| diagnosis: uti at admission |
| diagnosis: hiv_related at admission |
| diagnosis: measles at admission |
| diagnosis: varicella at admission |
| diagnosis: osteomyelitis at admission |
| diagnosis: febrile at admission |
| diagnosis: enteric_fever at admission |
| diagnosis: febrile at admission |
| diagnosis: epilepsy at admission |
| diagnosis: meningitis at admission |
| diagnosis: encephalopathy at admission |
| diagnosis: hydrocephalus at admission |
| diagnosis: developmentaldelay at admission |
| diagnosis: cerebral_palsy at admission |
| lumbar puncture at admission |
| fastbreathing at admission |
| grunting at admission |
| hypoxia at admission |
| respiratory at admission |
| conscious at admission |
| dehydration at admission |
| dysentery at admission |
| circulat at admission |
| rickets at admission |
| fever at admission |
| diarrhoea at admission |
| bglucose at admission |
| neuro at admission |
| sickle cell disease at admission |
| thalassaemia at admission |
| visualprob at admission |
| weight loss prior at admission |
| knowntb at admission |
| cough >14days at admission |
| known bcontact at admission |
| suspect tb at admission |
| hiv status at admission |
| on ART at admission |
| on ARVS at admission |
| hiv rdt at admission |
| pitc offered at admission |
| mother pmtct during delivery at admission |
| on cotrimoxazole at admission |
| hiv exposed |
| hiv status at admission |
| treated for SAM at admission |
| oedema at admission |
| oedema at discharge |
| haz at admission |
| waz at admission |
| wlz at admission |
| haz at discharge |
| waz at discharge |
| wlz at discharge |
| Left against advice at discharge |
| absconded |
| abnormal brith: premature |
| abnormal brith: less25kg |
| abnormal brith: twins |
| abnormal brith: bornterm |
| abnormal brith: unknown |
| temperature at discharge |
| absconded at discharge |
| respiratory rate at discharge |
| heart rate at discharge |
| oxygen staturations taken at discharge |
| oxygen saturations at discharge |
| airway at discharge |
| breathing: normal at discharge |
| breathing: centralcyanosis at discharge |
| breathing: nasalflaring at discharge |
| breathing: reducedairentry at discharge |
| breathing: wheeze at discharge |
| breathing: acidotic breathing at discharge |
| breathing: grunting at discharge |
| breathing: lower chest wall indrawing at discharge |
| breathing: crackles at discharge |
| breathing: dull to percussion at discharge |
| breathing at discharge |
| capillary refill at discharge |
| cold peripheries at discharge |
| avpu at discharge |
| fontanelle at discharge |
| tone at discharge |
| posture at discharge |
| activity at discharge |
| sunken eyes at discharge |
| skin pinch at discharge |
| breastfeeding at discharge |
| abdominal exam: normal at discharge |
| abdominal exam: distension at discharge |
| abdominal exam: hepatomegaly at discharge |
| abdominal exam: tendernes at discharge |
| abdominal exam: splenomegaly at discharge |
| abdominal exam: otherabdominalmass at discharge |
| rickets signs: none at discharge |
| rickets signs: wristwidening at discharge |
| rickets signs: rachiticrosary_disc |
| rickets signs: swollenknees at discharge |
| rickets signs: bowlegs at discharge |
| rickets signs: frontalbossing at discharge |
| jaundice at discharge |
| head & neck exam: mouthnormal at discharge |
| head & neck exam: earsnormal at discharge |
| head & neck exam: eyesnormal at discharge |
| head & neck exam: oralulceration at discharge |
| head & neck exam: pusfromear at discharge |
| head & neck exam: conjunctivitis at discharge |
| head & neck exam: oralcandidiasis at discharge |
| head & neck exam: tender swelling behind earat discharge |
| head & neck exam: eyedischarge at discharge |
| head & neck exam: stomatitis at discharge |
| head & neck exam: lymphadenopathy at discharge |
| head & neck exam: visualimpairmen at discharge |
| skin: normal at discharge |
| skin: hyperpigmen at discharge |
| skin: depigmen at discharge |
| skin: brokenskin at discharge |
| skin: dermatitis at discharge |
| skin: flakypaint at discharge |
| skin: cellulitis at discharge |
| skin: impetigo at discharge |
| skin: pustules at discharge |
| skin: vesicles at discharge |
| skin: esquamation at discharge |
| skin: macularpapular at discharge |
| rash site: trunk at discharge |
| rash site: facescalp at discharge |
| rash site: legs at discharge |
| rash site: palmssoles at discharge |
| rash site: buttocks |
| rash site: arms at discharge |
| rash site: perineum at discharge |
| rash site: na at discharge |
| nutrition program at discharge |
| currently breastfeeding at discharge |
| other at discharge |
| relactation input at discharge |
| relactation successful at discharge |
| breastfeeding counselling at discharge |
| relactation followup at discharge |
| nutrition counselling at discharge |
| exclusive breastfeeding at discharge |
| breastfeeding sufficient at discharge |
| referred for therapeutic feeding at discharge |
| refered suppllmentary feeding at discharge |
| clinician impression of risk at discharge |
| diagnosis: lrti at discharge |
| diagnosis: bronchiolitis at discharge |
| diagnosis: urti at discharge |
| diagnosis: pulmonarytb at discharge |
| diagnosis: otitismedia at discharge |
| diagnosis: asthma at discharge |
| diagnosis: anaemia at discharge |
| diagnosis: sickle cell disease at discharge |
| diagnosis: renal impairment at discharge |
| diagnosis: nephrotic syndrome at discharge |
| diagnosis: nephritis at discharge |
| diagnosis: liver dysfunction at discharge |
| diagnosis: congenital cardiac disease |
| diagnosis: gastroenteritis at discharge |
| diagnosis: sepsis at discharge |
| diagnosis: malaria at discharge |
| diagnosis: extrapulmonary tb at discharge |
| diagnosis: soft tissue infection at discharge |
| diagnosis: uti at discharge |
| diagnosis: hiv related illness at discharge |
| diagnosis: measles at discharge |
| diagnosis: varicella at discharge |
| diagnosis: osteomyelitis at discharge |
| diagnosis: febrile illness unspecified at discharge |
| diagnosis: confirmed enteric fever at discharge |
| diagnosis: febrile convulsions at discharge |
| diagnosis: epilepsy at discharge |
| diagnosis: lp confirmed meningitis at discharge |
| diagnosis: other encephalopathy at discharge |
| diagnosis: hydrocephalus at discharge |
| diagnosis: developmental delay at discharge |
| diagnosis: cerebralpalsy at discharge |
| diagnosis: congenital syndrome at discharge |
| length of stay |
| **DEMOGRAPHIC** |
| sex at admission |
| africa site |
| urban site |
| site |
| age in months at admission |
| population density near home |
| wasting prevalence near home |
| underweight prevalaence near home |
| stunting prev near home |
| distance to nearest hospital to home |
| distance to study hospital |
| **LABORATORY** |
| sodium at admission |
| potassium at admission |
| calcium at admission |
| albumin at admission |
| alt at admission |
| alk_phosphate at admission |
| urea at admission |
| creatinine at admission |
| bilirubin at admission |
| inorganic phosphate at admission |
| magnesium at admission |
| adjusted calcium at admission |
| red blood cells at admission |
| white blood cells at admission |
| platelets at admission |
| lymphocytes at admission |
| haemoglobin (altitude adjusted) at admission |
| sodium at discharge |
| potassium at discharge |
| calcium at discharge |
| albumin at discharge |
| alt at discharge |
| alkaline phosphate at discharge |
| urea at discharge |
| creatinine at discharge |
| bilirubin at discharge |
| inorganic phosphate at discharge |
| magnesium at discharge |
| adjusted calcium at discharge |
| haemoglobin at discharge |
| rbc at discharge |
| wbc at discharge |
| platelets at discharge |
| neutrophils at discharge |
| lymphocytes at discharge |
| osinophils at discharge |
| monocytes at discharge |
| basophils at discharge |
| haemoglobin (altitude adjested) at discharge |
| **SOCIAL** |
| brought by: mother at admission |
| brought by: father at admission |
| brought by: grandparent at admission |
| brought by: aunt at admission |
| brought by: sibling_less18 at admission |
| brought by: sibling_more18 at admission |
| brought by: carer at admission |
| brought by: other at admission |
| biologial parentsare related |
| carer present before admission |
| carer present |
| primary carer |
| marital status of caregiver |
| carer able to read |
| carer resp provider |
| lives with primary carer |
| childok pcurr comm |
| interviewee |
| child insured |
| reason for attending: reffered by health worker |
| reason for attending: caregiver concern |
| reason for attending: received money for transport |
| reason for attending: caregiver returned home |
| reason for attending: relative concern |
| reason for attending: other |
| means of travel to hospital: car |
| means of travel to hospital: bus |
| means of travel to hospital: motorbike |
| means of travel to hospital: walking |
| means of travel to hospital: tuktuk |
| means of travel to hospital: rickshaw |
| means of travel to hospital: train |
| means of travel to hospital: ambulance |
| means of travel to hospital: other |
| hospital travel time |
| hospital travel cost |
| preadmission medical care: notreatment |
| preadmission medical care: shop |
| preadmission medical care: hospital |
| preadmission medical care: dispensary |
| preadmission medical care: traditional_healer |
| preadmission medical care: pharmacy |
| preadmission medical care: private |
| preadmission medical care: herbalist |
| preadmission medical care: homeopathist |
| preadmission medical care: other |
| birth weight known |
| birth location |
| delivery mode: normal |
| delivery mode: assisted |
| delivery mode: caesarean |
| delivery_mode at admissionitted_neonatalu |
| delivery_mode at admissionitted_hospital_ |
| delivery mode: unknown |
| mother age at firstpreg |
| mother age now |
| birth order known |
| mother total live births |
| childs primary carer |
| biological father alive |
| biological mother alive |
| primary carer: age |
| primary carer: sex |
| primary carer: weight |
| primary carer: muac |
| primary carer: height |
| primary carer: educ |
| Caregiver hiv status in last 6months |
| mother unwell |
| mother died |
| birth weight |
| father unwell |
| father died |
| othwe carer unwell |
| othwe carer died |
| mother pregnant |
| mother birthed |
| othwe carer pregnant |
| othcarer birthed |
| primary carer work type |
| child in good health before illess |
| illness duration |
| illness duration weeks |
| drinking water source |
| toilet type |
| toilet shared |
| floor type |
| wall type |
| roof type |
| main cooking fuel |
| owns livestock |
| owns land |
| has bank acc ount |
| has electricity |
| has radio |
| has tv |
| has comp |
| has refridgeratory |
| hhold owns: watch |
| hhold owns: standardphone |
| hhold owns: smartphone |
| hhold owns: no |
| hhold owns: unknown |
| hhold owns: cart |
| hhold owns: bike |
| hhold owns: mcycle |
| hhold owns: car |
| hhold owns: boat |
| little interest PHQ-9 |
| feel depressed PHQ-9 |
| sleep trouble PHQ-9 |
| tiredness PHQ-9 |
| poor appetite PHQ-9 |
| failure feel PHQ-9 |
| concentration PHQ-9 |
| unstable move PHQ-9 |
| suicidal feel PHQ-9 |
| problem effects daily life PHQ-9 |
| if prim.carer working |
| if prim.carer working: accompies caregiver |
| if prim.carer working: nosubstitute care |
| if prim.carer working: unclear |
| if prim.carer working: in care home |
| if prim.carer working: mother |
| if prim.carer working: father |
| if prim.carer working: sibling < 18yrs |
| if prim.carer working: > 18 |
| if prim.carer working: grandparent |
| if prim.carer working: aunt/uncle/cousin |
| if prim.carer working: daycare out of home |
| if prim.carer working: daycare at home |
| drinkin water other |
| general water source |
| general water other |
| water fetch time known |
| water fetch time |
| weeks water available |
| water treatment: none |
| water treatment: bleach |
| water treatment: strainthro_cloth |
| water treatment: letitstand_settle |
| water treatment: use_water_filter |
| water treatment: solar_disinfection |
| water treatment: boil |
| water treatment: other |
| number sharing toilet |
| number of toilets shared |
| toilet location |
| sleeping rooms |
| floor type: other |
| wall type: other |
| roof type: other |
| cook fuel: other |
| has kitchen |
| cook area location |
| number of cows |
| number of sheep |
| number of equine |
| number of goats |
| number of poult |
| number of other |
| acreage known |
| number of acres |
| missed meal in last 7 days |
| worry about food in last week |
| no prefered food in last week |
| limited preferred food in last week |
| some preferred food in last week |
| few meals in last week |
| hunger at nighte in last week |
| hunger for days in last week |
| How is food given to the child |
| typical food: milkproducts |
| typical food: breastmilk |
| typical food: cerealproducts |
| typical food: seafoods |
| typical food: root_tubers |
| typical food: vegetables |
| typical food: fruits |
| typical food: meat_poultry |
| typical food: eggs |
| typical food: pulses_seeds |
| typical food: fats_oils |
| typical food: sugars_juices |
| typical food: unknown |

**APPENDIX 2: Additional data from parent cohort**

**Inverse probability weights:** Because of the non-proportional stratified sampling in this study, we created sampling weights (3 weights for the 3 strata) proportion to inverse of sampling fraction of the respective group from a typical hospital admission in Africa and south Asia. Hospital Paediatric admission surveillance data (for children 2 to 23 months old) during the period of CHAIN study from four site hospitals were used to estimate the mean proportions across the three nutrition strata. There was no ongoing Paediatric admission surveillance in the other five sites. Using the estimated proportions and the actual proportion of children recruited in CHAIN study we calculated the inverse probability of a children being recruited in each nutrition strata and standardized the inverse probability by diving with the NW group probability (**Table S2**).

| **Table 1. Sampling inverse weights.** | | | | | | | | | |
| --- | --- | --- | --- | --- | --- | --- | --- | --- | --- |
|  | Proportions admitted in each nutrition strata in CHAIN sites | | | | | Proportion recruited to CHAIN | Proportion of CHAIN enrolments to hospital admissions | Inverse probability weights (1/proportion of CHAIN enrolments to hospital admissions | Inverse selection weights (standardized by NW) |
|  | Kilifi | Migori | Banfora | Dhaka* | Average |  |  |  |  |
| Not wasted | 65% | 56% | 58% | 57% | 59% | 36·1% | 0·61 | 1·63 | 1 |
| Moderately wasted | 13% | 17% | 16% | 19% | 16% | 24·6% | 1·54 | 0·65 | 0·40 |
| Severely wasted or Kwashiorkor | 22% | 27% | 26% | 24% | 25% | 39·3% | 1·57 | 0·64 | 0·39 |
| *Defined using weight-for-length z-scores, other sites were defined using low MUAC according to the study enrolment criteria | | | | | | | | | |

**Overlap between 30-day and post-discharge:** There were 54 children who died within 30-days of enrollment but in the post-discharge period. These children are therefore included in both analyses. The investigators consider the 30-day and the 180-day post-discharge models to answer separate questions, and therefore we retain these 54 children in both models.

**Loss to follow-up**: Of 3101 children enrolled vital status at day 180 was available for 2,985 children (96.5%).

**Missing data:** Missing data are not excluded from extreme gradient boosted models, but are used as a potential predictor of the outcome.

| **Variable** | **Proportion missing N (%)** |
| --- | --- |
| Admission biochemistry | 273 (8·8) |
| Discharge biochemistry^1^ | 626 (21·7%) |
| Admission complete blood count | 172 (5·6) |
| Discharge complete blood count^1^ | 573 (19·8%) |
| HIV | 76 (2·5) |
| Admission Blood glucose | 59 (1·9) |
| Admission height-for-age z-scores | 10 (0·3) |
| Birth size^a^ | 40 (1·4) |
| Recommended appropriate diet^a^ | 10 (0·4) |
| Travel cost^a^ | 56 (1·9) |
| Household GPS coordinates | 3 (0·1) |
| Population density | 3 (0·1) |
| Water availability^2^ | 6 (0·2) |
| Type of toilet^2^ | 1 (0.03) |
| Mother mental health^2^ | 34 (1·2) |
| Mother sick^2^ | 13 (0·5) |
| Mother working^2^ | 30 (1·0) |
| Caregiver education level^2^ | 17 (0·6) |
| Biological mother as primary caregiver | 17 (0·6) |

^1^Missing discharge information is only among children discharged alive.

^2^These variables were excluded from the 30 day analysis. Missingness is displayed for the discharge analysis alone.

**APPENDIX 3: Supplementary Tables & Figures**

**Supplementary Table 1:** Participants selected characteristics at discharge from index admission.

|  | **NW (N=1072)** | **MW (N=724)** | **SWK  (N=1078)** |
| --- | --- | --- | --- |
| Demographics |  |  |  |
| Age — months median (interquartile range) | 11·4 (7·5−16·3) | 10·9 (7·3−14·8) | 10·7 (6·41−16·2) |
| Gender (female) — no. (%) | 410 (38) | 317 (44) | 512 (48) |
| Abnormal discharge — no. (%) | 60 (5·6) | 39 (5·4) | 106 (9·8) |
| Hospital admission duration (days) median (IQR)% | 3 (2−5) | 4 (2−6) | 7 (4−12) |
| Change in undernutrition at discharge — no. (%)^+^ |  |  |  |
| No change | 1034 (96) | 599 (83) | 814 (76) |
| Improved | 0 | 90 (12) | 264 (24) |
| Worsened | 38 (3·5) | 35 (4·8) | 0 |
| Acute illness at discharge |  |  |  |
| Systemic inflammatory response syndrome (SIRS) — no. (%) | 146 (14) | 98 (14) | 171 (16) |
| Severe Pneumonia — no. (%) | 61 (5·7) | 36 (5·0) | 70 (6·4) |
| Any sign of shock — no. (%) | 149 (14) | 91 (13) | 225 (21) |
| Neurological (AVPU >A) — no. (%) | 1 (0·09) | 2 (0·3) | 0 |
| Anemia — no. (%) |  |  |  |
| None | 151 (14) | 92 (13) | 114 (11) |
| Mild | 326 (30) | 170 (23) | 215 (20) |
| Moderate | 522 (49) | 405 (56) | 659 (61) |
| Severe | 73 (6·8) | 57 (7·8) | 90 (8·4) |
| Anthropometry |  |  |  |
| Nutritional edema — no. (%) | 1 (0·09) | 0 | 44 (4·1) |
| MUAC (CM) — mean (sd) | 13·6 ±1·0 | 12·0 ±0·4 | 10·8 ±1·3 |
| Weight-for-length z score — mean (sd)* | -0·5 ±1·1 | -2·1 ±1·0 | -2·8 ±1·4 |
| Weight-for-age z score — mean (sd)* | -1·1 ±1·1 | -2·6 ±1·0 | -3·8 ±1·3 |
| Length-for-age z score — mean (sd) | -1·2 ±1·3 | -2·0 ±1·3 | -3·2 ±1·6 |
| ^+^change in nutritional status at discharge was defined as no change (no change in the NW, MW, SWK groups), improved (moved from SWK to MW/NW or from MW to NW) and worsened (moved from NW to MW/SWK or from MW to SWK), *children with edema excluded from these values | | | |

**Supplementary Table 2: Children at risk and events for Kaplan Meier curves in manuscript.**

**30-day mortality**

| **Cluster** | **N** | **Day 10** | | **Day 20** | | **Day 30** | |
| --- | --- | --- | --- | --- | --- | --- | --- |
|  |  | **At Risk** | **Events** | **At Risk** | **Events** | **At Risk** | **Events** |
| **1** | 727 | 693 | 6 | 691 | 6 | 691 | 6 |
| **2** | 1188 | 1150 | 23 | 1146 | 25 | 1140 | 30 |
| **3** | 880 | 832 | 45 | 803 | 70 | 789 | 80 |
| **4** | 187 | 174 | 12 | 163 | 24 | 161 | 25 |
| **5** | 81 | 29 | 52 | 26 | 54 | 23 | 57 |
| **6** | 37 | 10 | 29 | 3 | 34 | 1 | 36 |

**Post-discharge mortality**

| **Cluster** | **N** | **Day 45** | | **Day 90** | | **Day 180** | |
| --- | --- | --- | --- | --- | --- | --- | --- |
|  |  | **At Risk** | **Events** | **At Risk** | **Events** | **At Risk** | **Events** |
| **A** | 1043 | 1026 | 1 | 1011 | 1 | 661 | 2 |
| **B** | 710 | 708 | 1 | 706 | 2 | 497 | 3 |
| **C** | 373 | 366 | 5 | 355 | 13 | 152 | 19 |
| **D** | 493 | 465 | 24 | 445 | 37 | 293 | 53 |
| **E** | 215 | 159 | 41 | 149 | 50 | 76 | 65 |
| **F** | 52 | 38 | 15 | 33 | 18 | 18 | 24 |

**Supplementary Figure 1: Top 50 predicators of 30-day mortality**

**
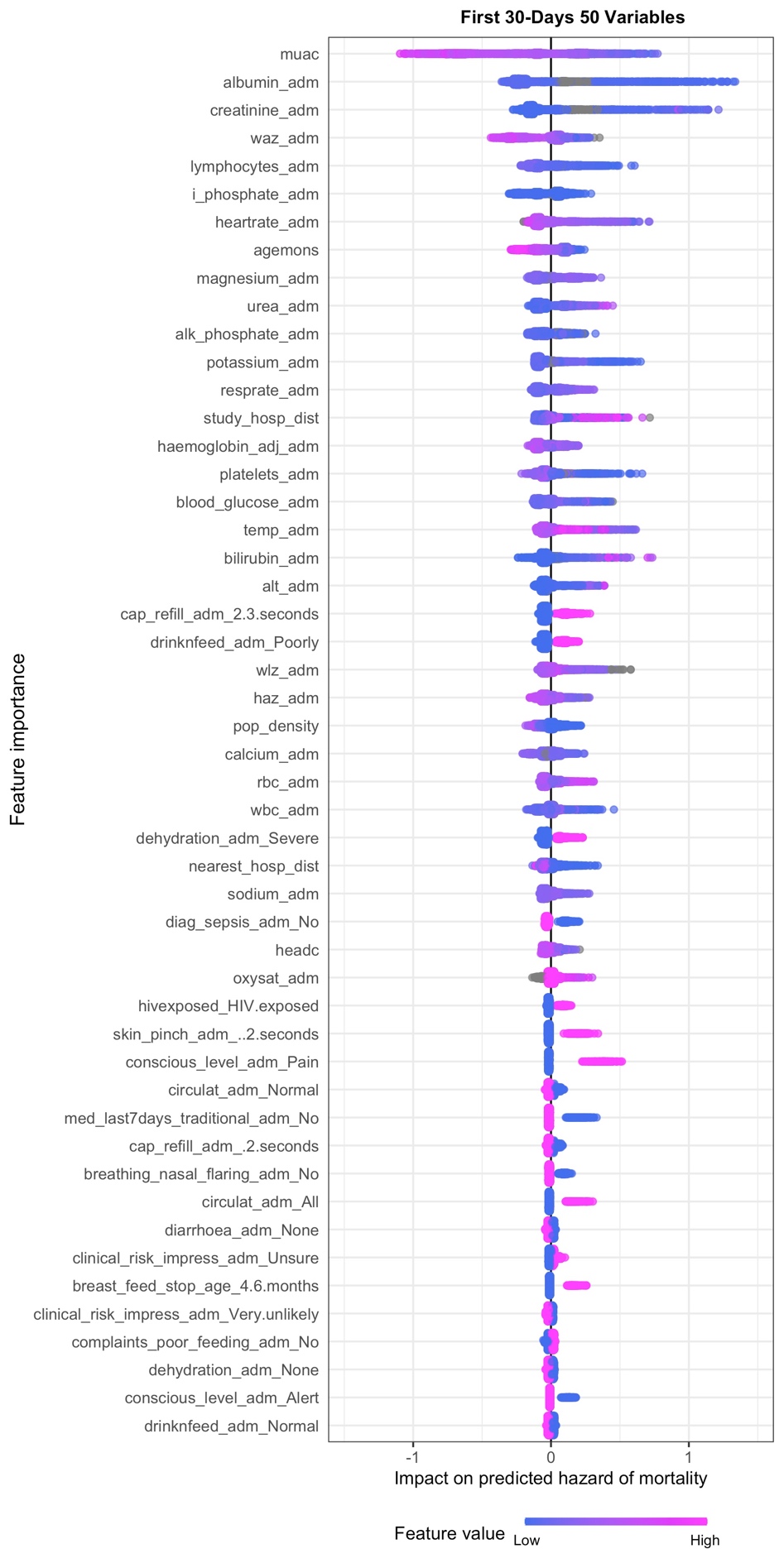
**

**Supplementary Figure 2: Alternative cluster numbers 30-day mortality results**

**
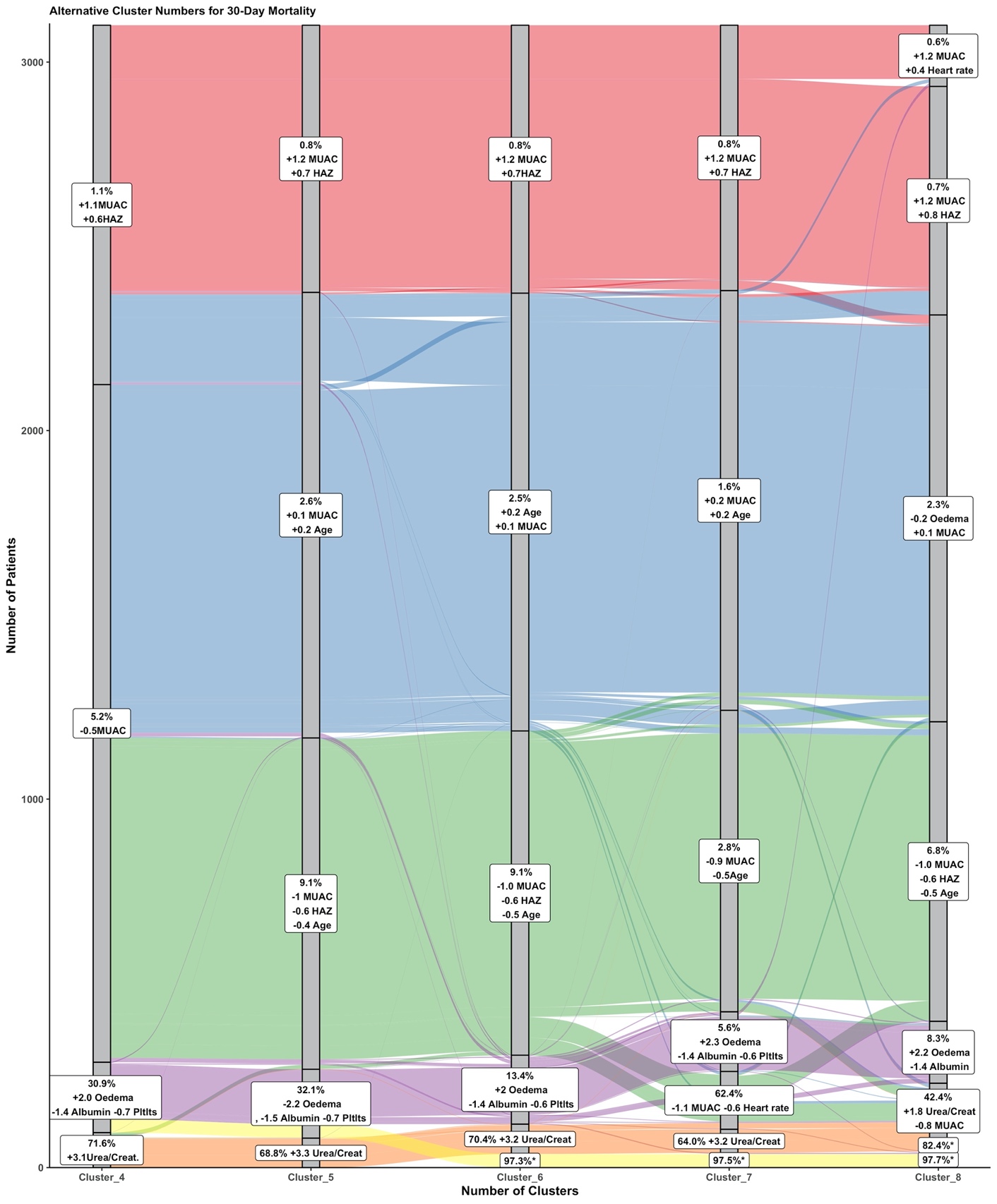
**

*for ease of view characteristics of some clusters have been removed: **six cluster model** - cluster F (yellow band, 97·3% mortality): +2·3 oedema, -1·3 albumin, -1·1 platelets, +0·7 temperature. **seven cluster model** - cluster G (yellow band, 97·5% mortality): +1·9 oedema, -1·5 albumin, -1·0 platelets, -0·9 heart rate. **eight cluster model** - cluster G (orange band, 82·4% mortality): 4·1 Creatinine, 3·7 Urea, -1·5 WLZ; cluster H (yellow band, 97·7% mortality): +1·9 oedema, -1·5 albumin, -1·0 platelets, -0·9 heart rate.

**Supplementary Figure 4a: Four and five cluster variants of results for 30-day mortality.**

**
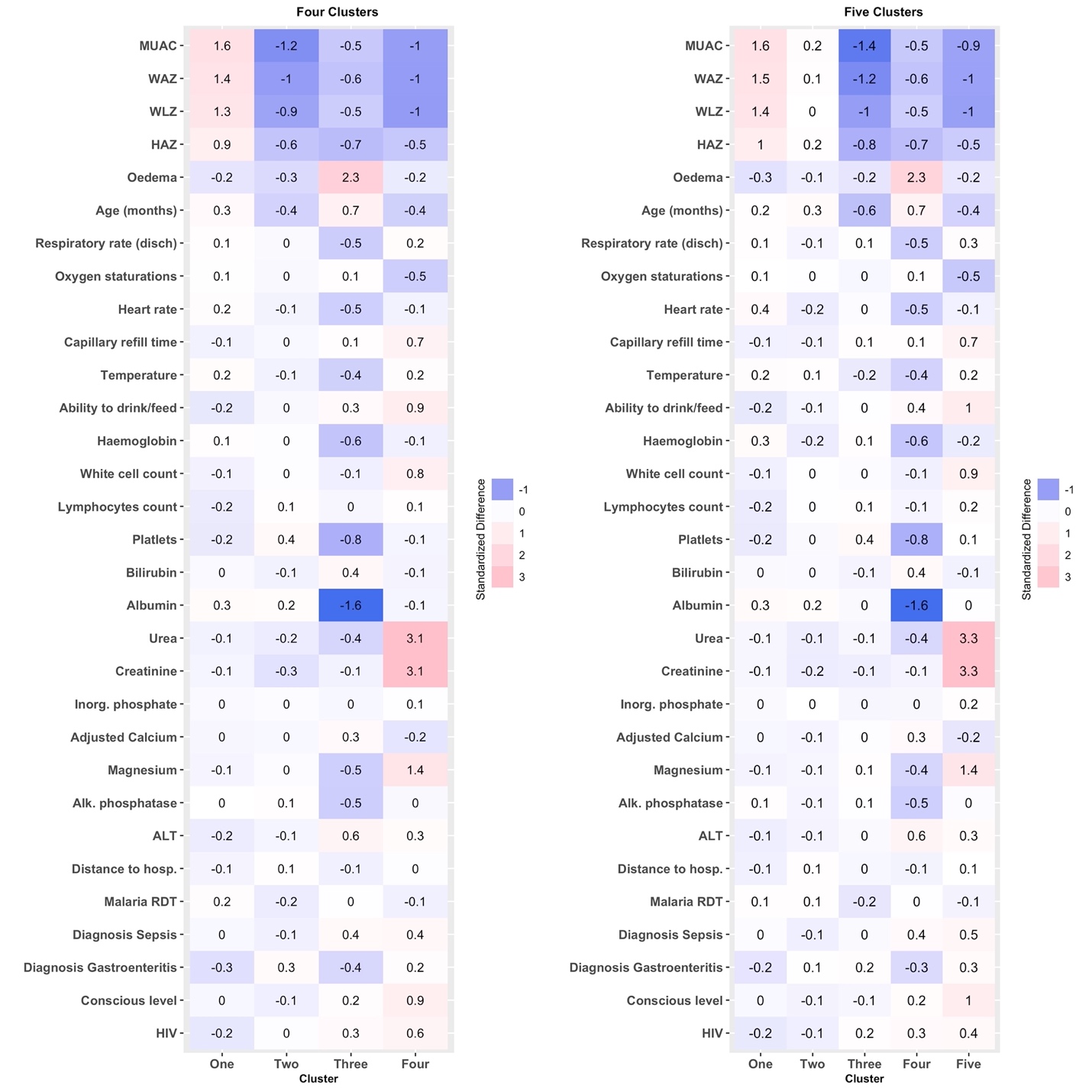
**

**Supplementary Figure 4b: Seven and eight cluster variants of the results for 30-day mortality (six cluster variant in main text)**

**
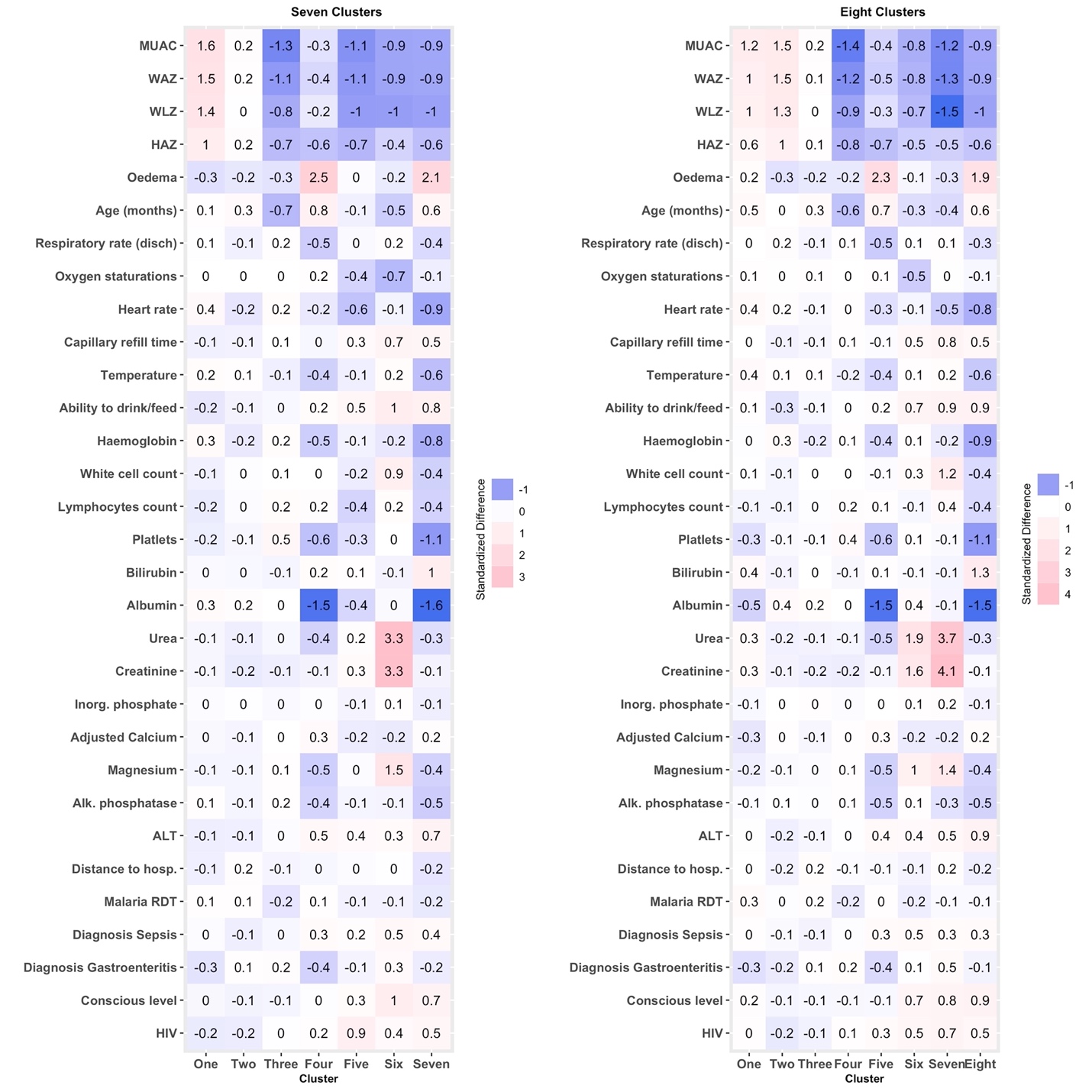
**

**Supplementary Figure 5: Top 50 180-day results**

**
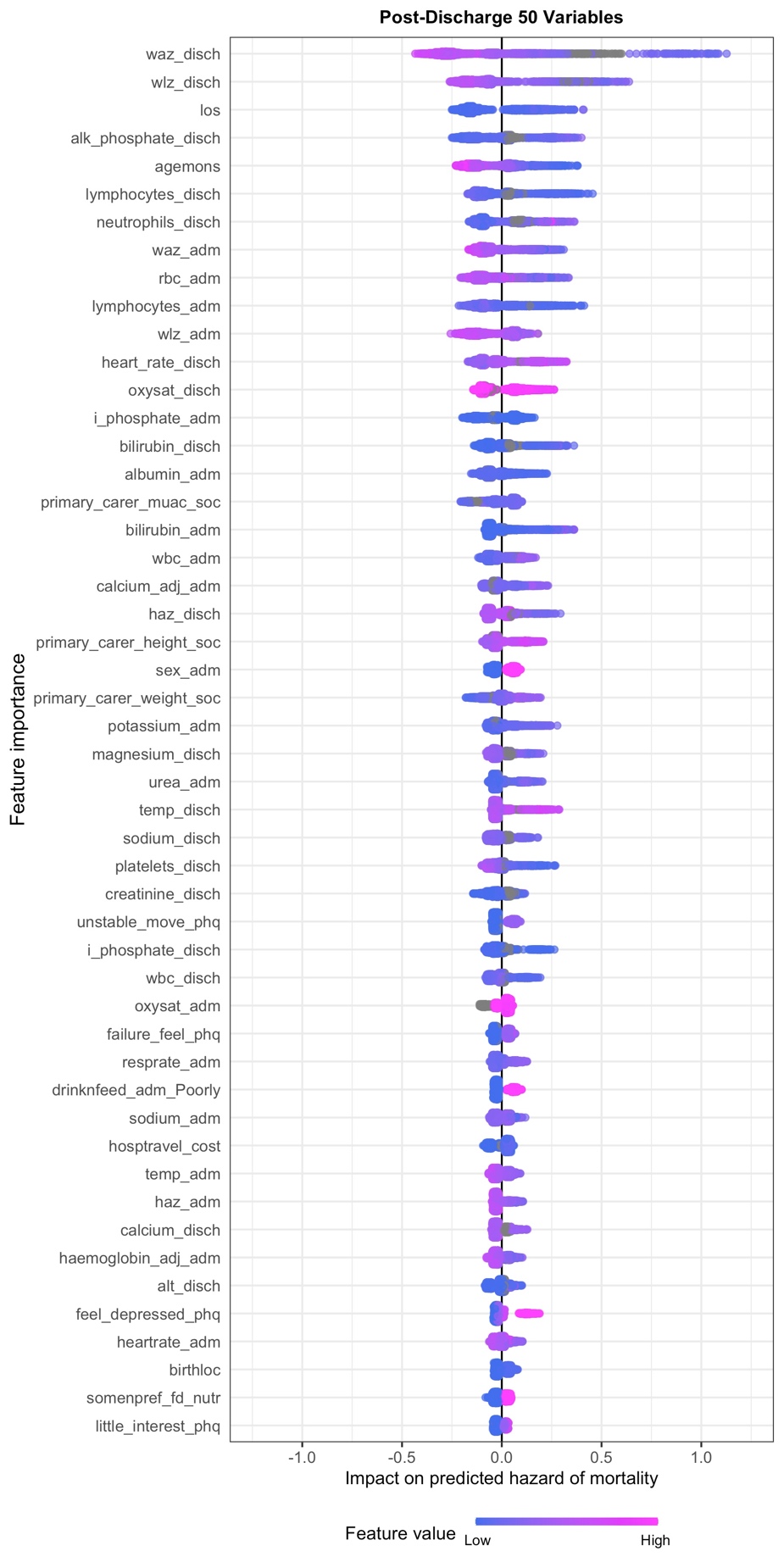
**

**Supplementary Figure 6: Phenotypes of Post-discharge mortality.** The color and value of each cell represents the standardize difference between the mean of the cluster and the total sample mean for the relevant variable, i.e. (Mean_clusterX_ – Mean_allotherclusters_)/Standard Deviation_sample_.

**
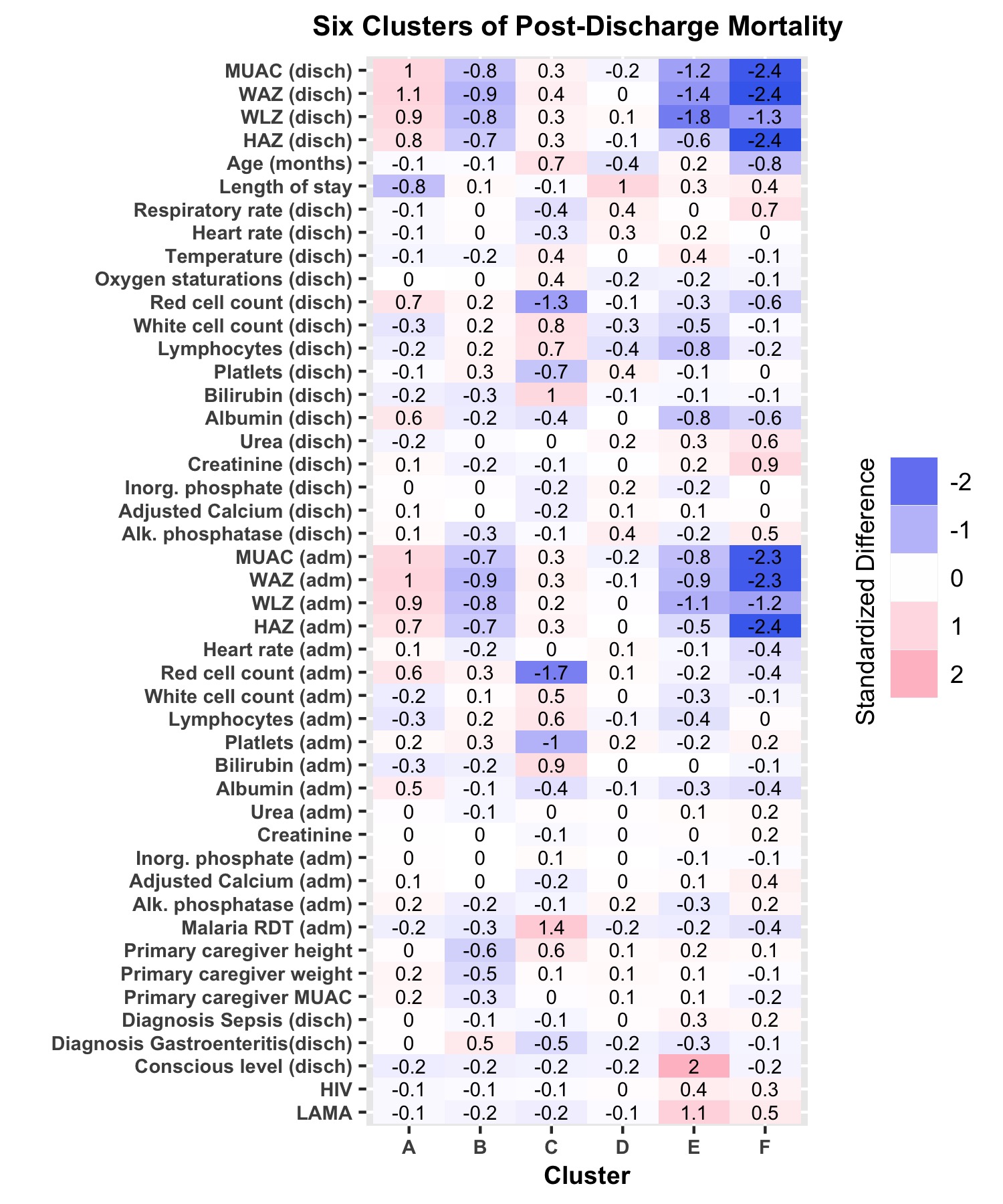
**

Foot note: For continuous variables a value above 0 in this table is indicative of a higher mean value in that variable, e.g. +0·9 urea suggest that cluster has a mean urea 0.9 SD higher that the other clusters.

**Supplementary Figure 7: Alternative cluster numbers 180-day mortality results**


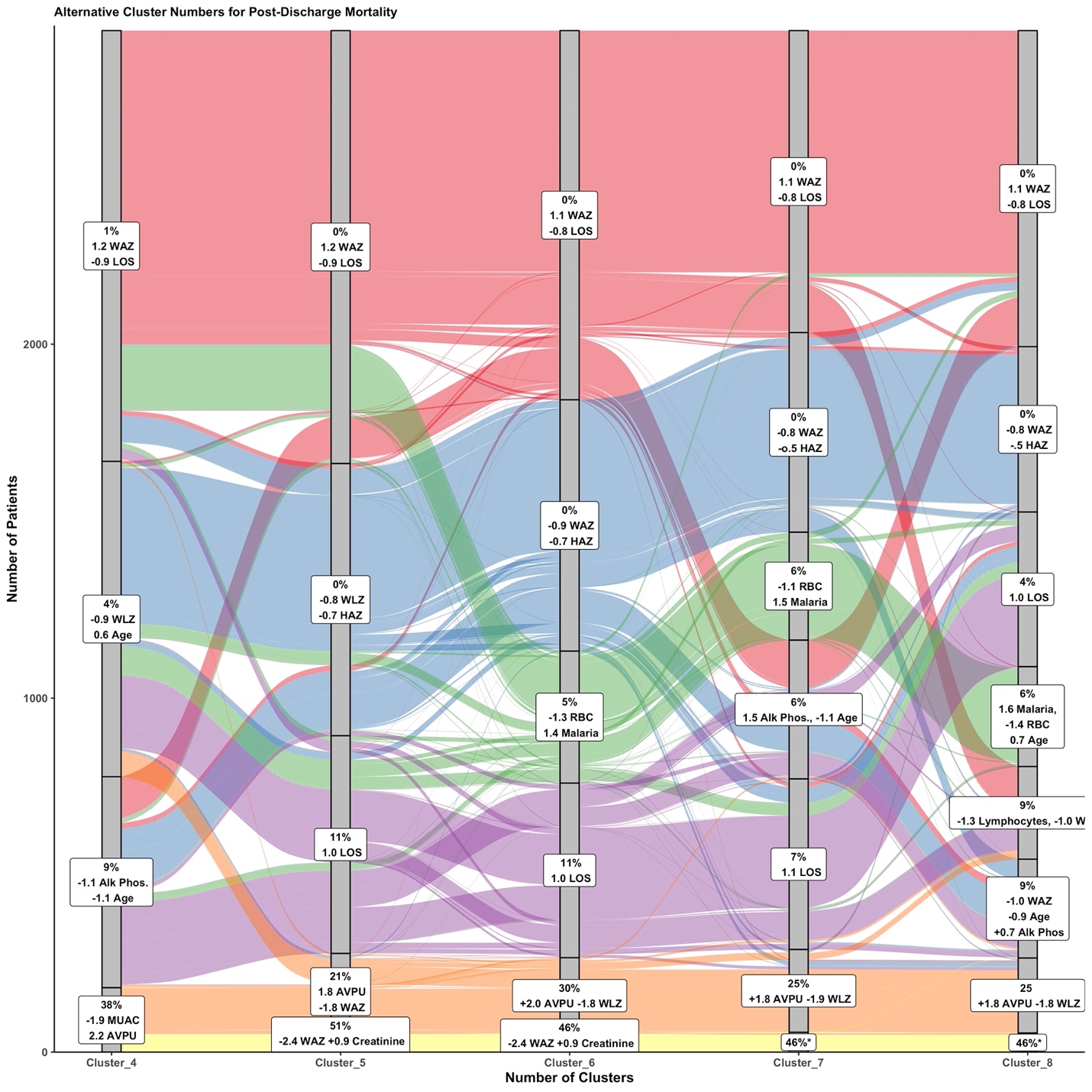


*for ease of view characteristics of some clusters have been removed: **seven cluster model** - cluster G (yellow band, 46% mortality): -2·4 MUAC at discharge, -2·4 HAZ at discharge, -0·9 creatinine. **eight cluster model** - cluster H (yellow band, 46% mortality): -2·4 MUAC at discharge, -2·4 HAZ at discharge, -1·0 creatinine.

**Supplementary Figure 8a: Four & five cluster variants of the results for post-discharge mortality.**


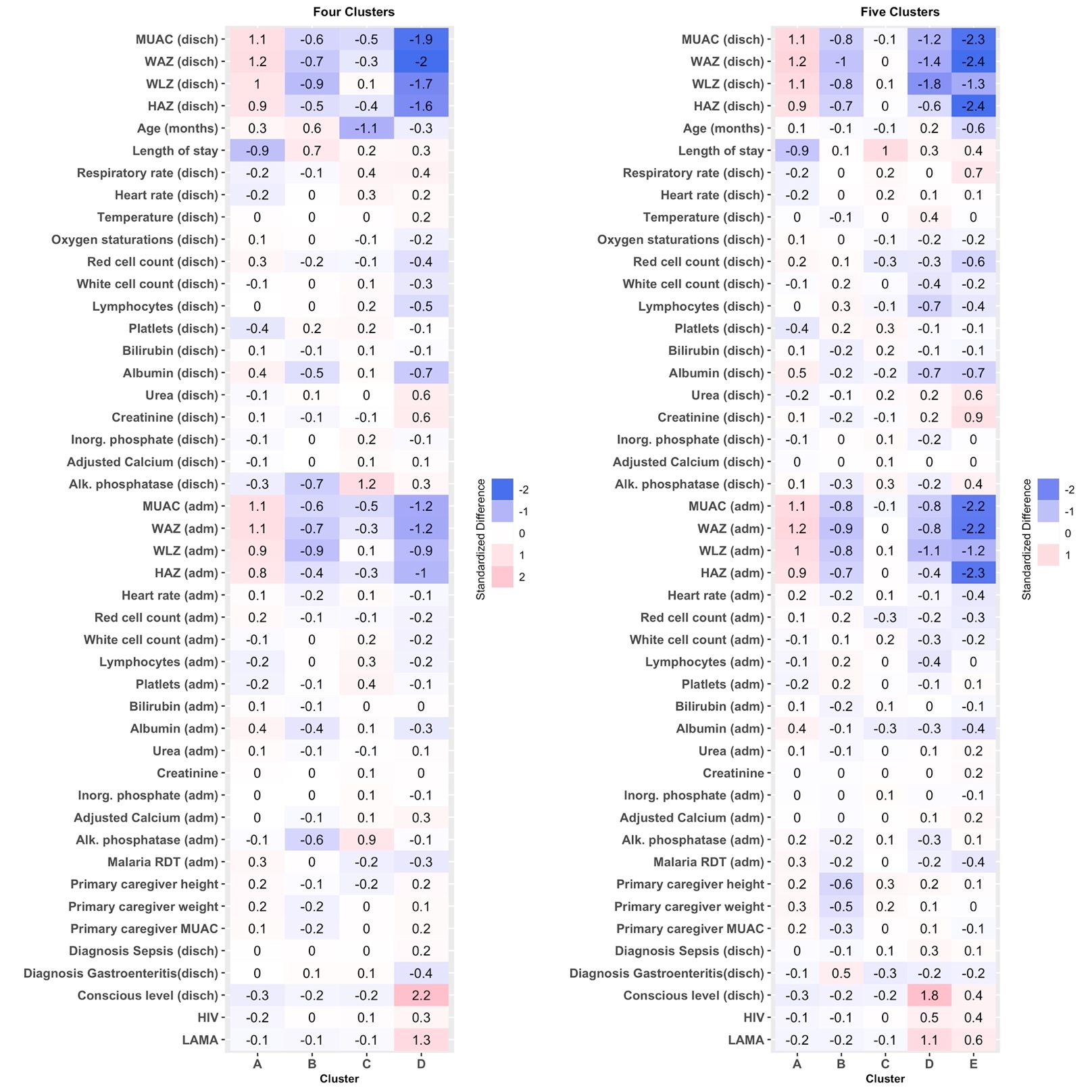


**Supplementary Figure 8b: Seven eight cluster variant of results for post-discharge mortality**


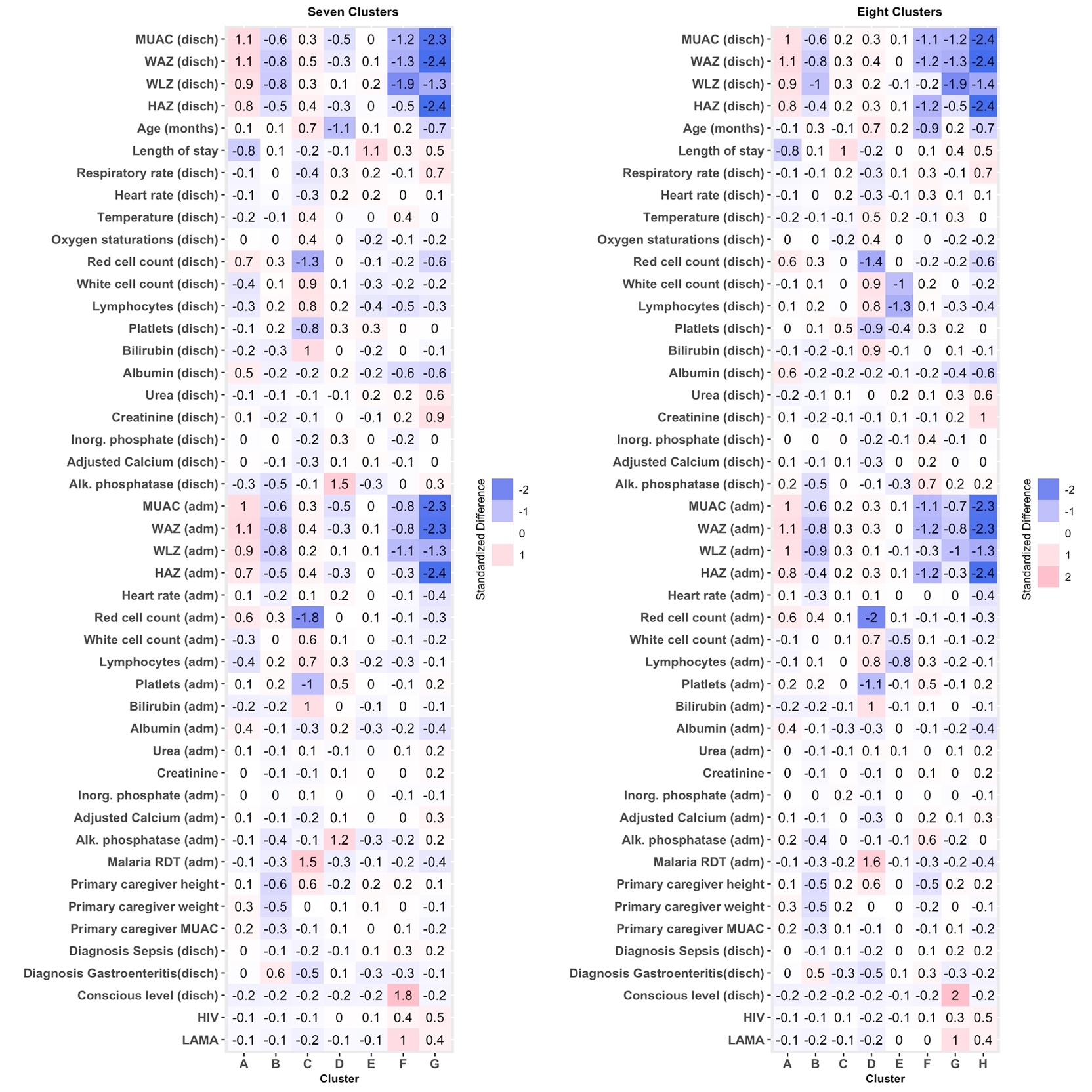


**Supplementary Figure 9a: Predictive performance of the 30-day models with a reducing number of included variables.**

**
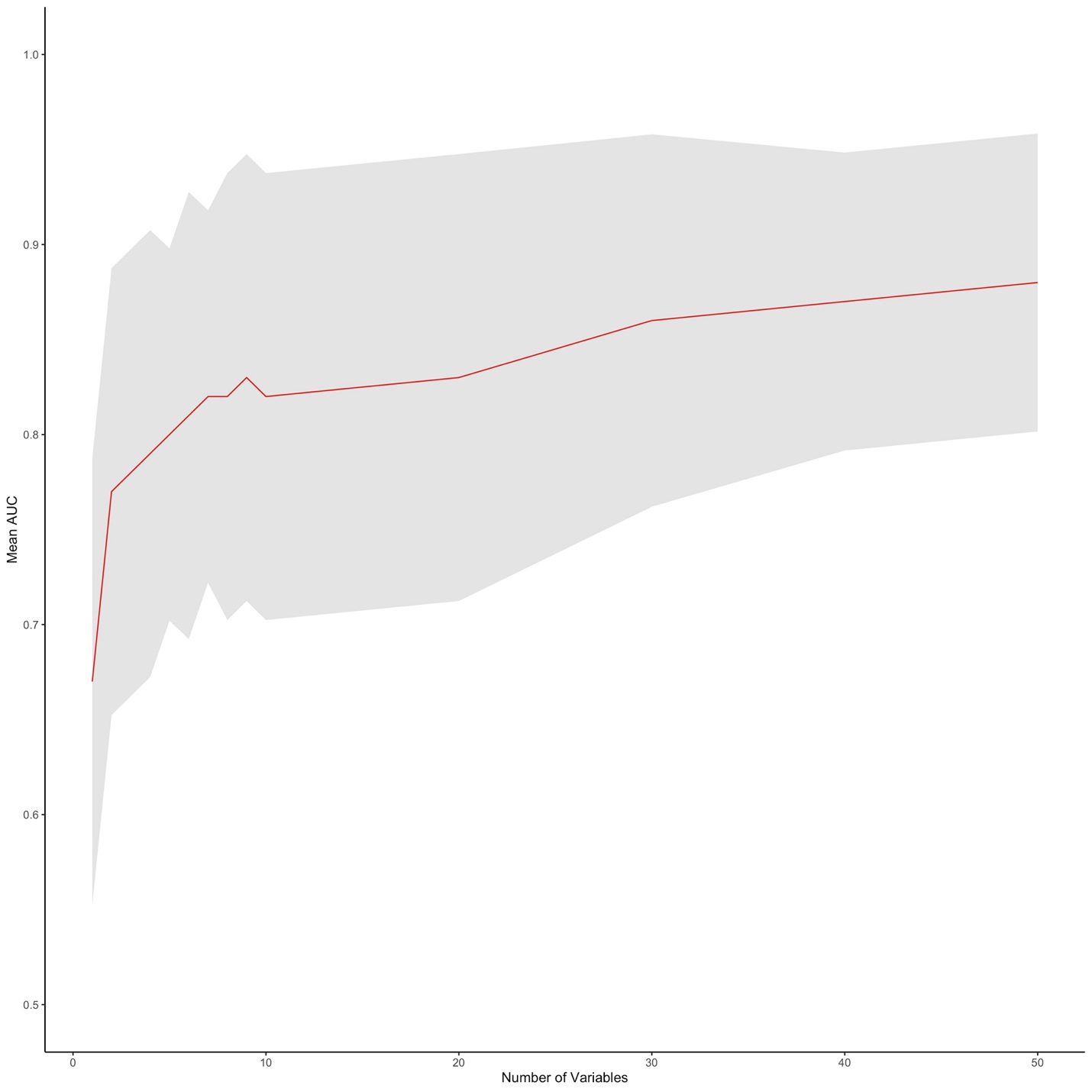
**

**Supplementary Figure 9b: Predictive performance of the post-discharge models with a reducing number of included variables.**


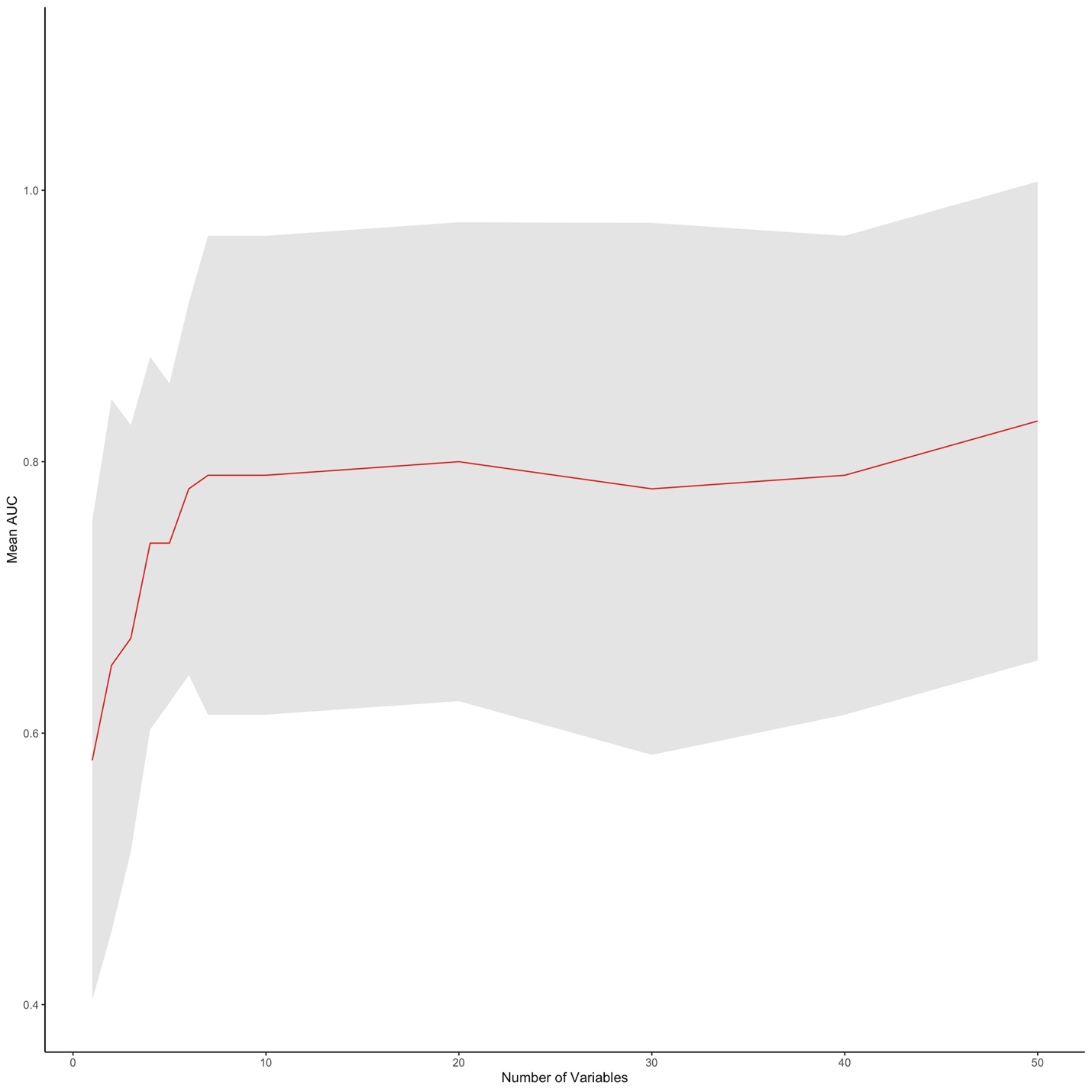


**Appendix 4: CHAIN Co-Author Name List for PubMed (alphabetical order)**

| **First/ Middle Name(s)/Initial(s)** | **Last Name** | **Initials** |
| --- | --- | --- |
| Abdoulaye Hama | Diallo | AHD |
| Abu Sadat Mohammad | Sayeem Bin Shahid | AMS |
| Ali Fazal | Khan | AFK |
| Ali Faisal | Saleem | AFS |
| Benson O. | Singa | BOS |
| Blaise Siezanga | Gnoumou | BSG |
| Caroline | Tigoi | CT |
| Catherine Achieng | Otieno | CAO |
| Celine | Bourdon | CB |
| Chris Odhiambo | Oduol | COO |
| Christina L. | Lancioni | CLL |
| Christine | Manyasi | CMan |
| Christine J. | McGrath | CJM |
| Christopher | Maronga | CMar |
| Christopher | Lwanga | CL |
| Daniella | Brals | DB |
| Dilruba | Ahmed | DA |
| Dinesh | Mondal | DM |
| Donna M. | Denno | DMD |
| Dorothy I. | Mangale | DIM |
| Emmanuel | Chimezi | EC |
| Emmie | Mbale | EMb |
| Ezekiel | Mupere | EMu |
| Gazi Md. Salauddin | Mamun | GSM |
| Issaka | Ouedraogo | IO |
| George | Githinji | GG |
| James A. | Berkley | JAB |
| Jenala | Njirammadzi | JN |
| John | Mukisa | JM |
| Johnstone | Thitiri | JT |
| Jonas | Haggstrom | JH |
| Joseph D. | Carreon | JDC |
| Judd L. | Walson | JLW |
| Julie | Jemutai | JJ |
| Kirkby D. | Tickell | KDT |
| Lubaba | Shahrin | LS |
| MacPherson | Mallewa | MMa |
| Md. Iqbal | Hossain | MH |
| Mohammod Jobayer | Chisti | MJC |
| Molly | Timbwa | MT |
| Moses | Mburu | MMb |
| Moses M. | Ngari | MMN |
| Narshion | Ngao | NN |
| Peace | Aber | PA |
| Philliness Prisca | Harawa | PPH |
| Priya | Sukhtankar | PS |
| Robert H. J. | Bandsma | RHB |
| Roseline Maimouna | Bamouni | RMB |
| Sassy | Molyneux | SMo |
| Sergey | Feldman | SF |
| Shalton | Mwaringa | SMw |
| Shamsun Nahar | Shaima | SNS |
| Syed Asad | Ali | SAA |
| Syeda Momena | Afsana | SMA |
| Syera | Banu | SB |
| Tahmeed | Ahmed | TA |
| Wieger P. | Voskuijl | WPV |
| Zaubina | Kazi | ZK |
