# Supplementary material for: Characterising paediatric mortality during and after acute illness in Sub-Saharan Africa and South Asia: a secondary analysis of the CHAIN cohort using a machine learning approach
Source: eClinicalMedicine. 2023 Feb 6;57:101838. doi: 10.1016/j.eclinm.2023.101838 (PMC9941052; doi:10.1016/j.eclinm.2023.101838)
Supplement: CHAIN_Co-Author_PubMed_Name_List [file mmc2.docx]

**CHAIN Co-Author Name List for PubMed (alphabetical order)**

| **First/ Middle Name(s)/Initial(s)** | **Last Name** | **Initials** |
| --- | --- | --- |
| Abdoulaye Hama | Diallo | AHD |
| Abu Sadat Mohammad | Sayeem Bin Shahid | AMS |
| Ali Fazal | Khan | AFK |
| Ali Faisal | Saleem | AFS |
| Benson O. | Singa | BOS |
| Blaise Siezanga | Gnoumou | BSG |
| Caroline | Tigoi | CT |
| Catherine Achieng | Otieno | CAO |
| Celine | Bourdon | CB |
| Chris Odhiambo | Oduol | COO |
| Christina L. | Lancioni | CLL |
| Christine | Manyasi | CMan |
| Christine J. | McGrath | CJM |
| Christopher | Maronga | CMar |
| Christopher | Lwanga | CL |
| Daniella | Brals | DB |
| Dilruba | Ahmed | DA |
| Dinesh | Mondal | DM |
| Donna M. | Denno | DMD |
| Dorothy I. | Mangale | DIM |
| Emmanuel | Chimezi | EC |
| Emmie | Mbale | EMb |
| Ezekiel | Mupere | EMu |
| Gazi Md. Salauddin | Mamun | GSM |
| Issaka | Ouedraogo | IO |
| George | Githinji | GG |
| James A. | Berkley | JAB |
| Jenala | Njirammadzi | JN |
| John | Mukisa | JM |
| Johnstone | Thitiri | JT |
| Jonas | Haggstrom | JH |
| Joseph D. | Carreon | JDC |
| Judd L. | Walson | JLW |
| Julie | Jemutai | JJ |
| Kirkby D. | Tickell | KDT |
| Lubaba | Shahrin | LS |
| MacPherson | Mallewa | MMa |
| Md. Iqbal | Hossain | MH |
| Mohammod Jobayer | Chisti | MJC |
| Molly | Timbwa | MT |
| Moses | Mburu | MMb |
| Moses M. | Ngari | MMN |
| Narshion | Ngao | NN |
| Peace | Aber | PA |
| Philliness Prisca | Harawa | PPH |
| Priya | Sukhtankar | PS |
| Robert H. J. | Bandsma | RHB |
| Roseline Maimouna | Bamouni | RMB |
| Sassy | Molyneux | SMo |
| Sergey | Feldman | SF |
| Shalton | Mwaringa | SMw |
| Shamsun Nahar | Shaima | SNS |
| Syed Asad | Ali | SAA |
| Syeda Momena | Afsana | SMA |
| Syera | Banu | SB |
| Tahmeed | Ahmed | TA |
| Wieger P. | Voskuijl | WPV |
| Zaubina | Kazi | ZK |
